# Supplementary material for: α-Synuclein aggregates induce mitochondrial damage and trigger innate immunity to drive neuron–microglia communication
Source: Nat Commun. 2026 May 15;17:6462. doi: 10.1038/s41467-026-73136-7 (PMC13376753; doi:10.1038/s41467-026-73136-7)

Supplementary Information

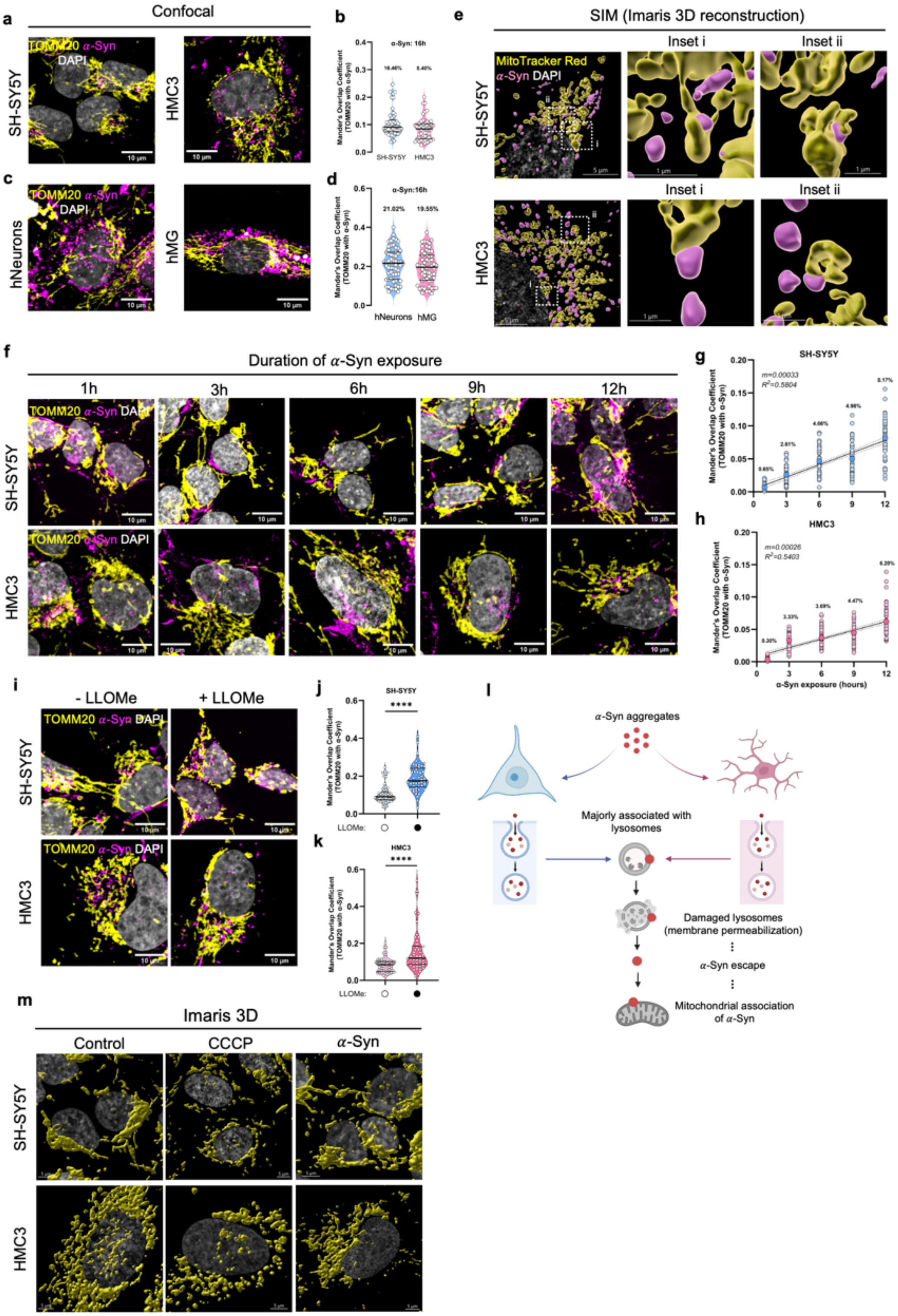

Supplementary Fig. 1. α-Syn localization with mitochondria, and morphological alterations. Representative confocal images of α-Syn overlap with TOMM20+ mitochondria in neuronal and microglial cells. (b) Quantification of Mander's overlap coefficient of fraction overlap between mitochondria and α-Syn. N=3 independent experiments, n=50 cells. (c) Representative confocal images of α-Syn overlap with TOMM20+ mitochondria in hiPSC-derived neurons (hNeurons) and microglia (hMG). (d) Quantification of Mander's overlap coefficient of fraction overlap between mitochondria and α-Syn. N=3 independent experiments, n=60 cells. (e) Super-resolution images depicting overlap of α-Syn and mitochondria in neuronal and microglial cells. (e) Representative 3D reconstructions of super-resolution SIM images highlighting α-Syn association with mitochondria in neuronal and microglial cells. (f) Time-course analysis of α-Syn association with mitochondria in neuronal and microglial cells. (g-h) Quantification of Mander's overlap co-efficient of fraction of TOMM20+ mitochondria overlapping with α-Syn per neuronal cell (g) and microglial cell (h). N=3 independent experiments, n=50 cells per time-point. Mean values for each time point are in bold, darker circles. Solid lines represent simple linear regression fit, with 95% confidence interval denoted by dashed lines. Mean percentage of overlap mentioned within the graphs. (i) Representative confocal images of neuronal cells (top panels) and microglial cells (bottom panels) treated or not with LLOMe to assess α-Syn localization with TOMM20+ mitochondria. (j-k) Quantification of Mander's overlap coefficient of fraction of TOMM20+ mitochondria overlapping with α-Syn after 16 hours of incubation in control and LLOMe-treated (for 1h) conditions in neuronal cells (j) and microglial cells (k). Control groups are same as in panel (b). N=3 independent experiments, n=50 cells. Statistical significance was analyzed using Mann-Whitney test. ****p<0.0001. Data represented as median and quartiles. (l) Schematic representation of lysosomal escape of α-Syn and subsequent mitochondrial association. (m) Imaris 3D reconstruction of TOMM20+ mitochondria in control, CCCP-treated, and α-Syn-exposed conditions for neuronal and microglial cells. Schematic in panel (l) was created in BioRender. MAYA, S. (2026) https://BioRender.com/54gaiqn

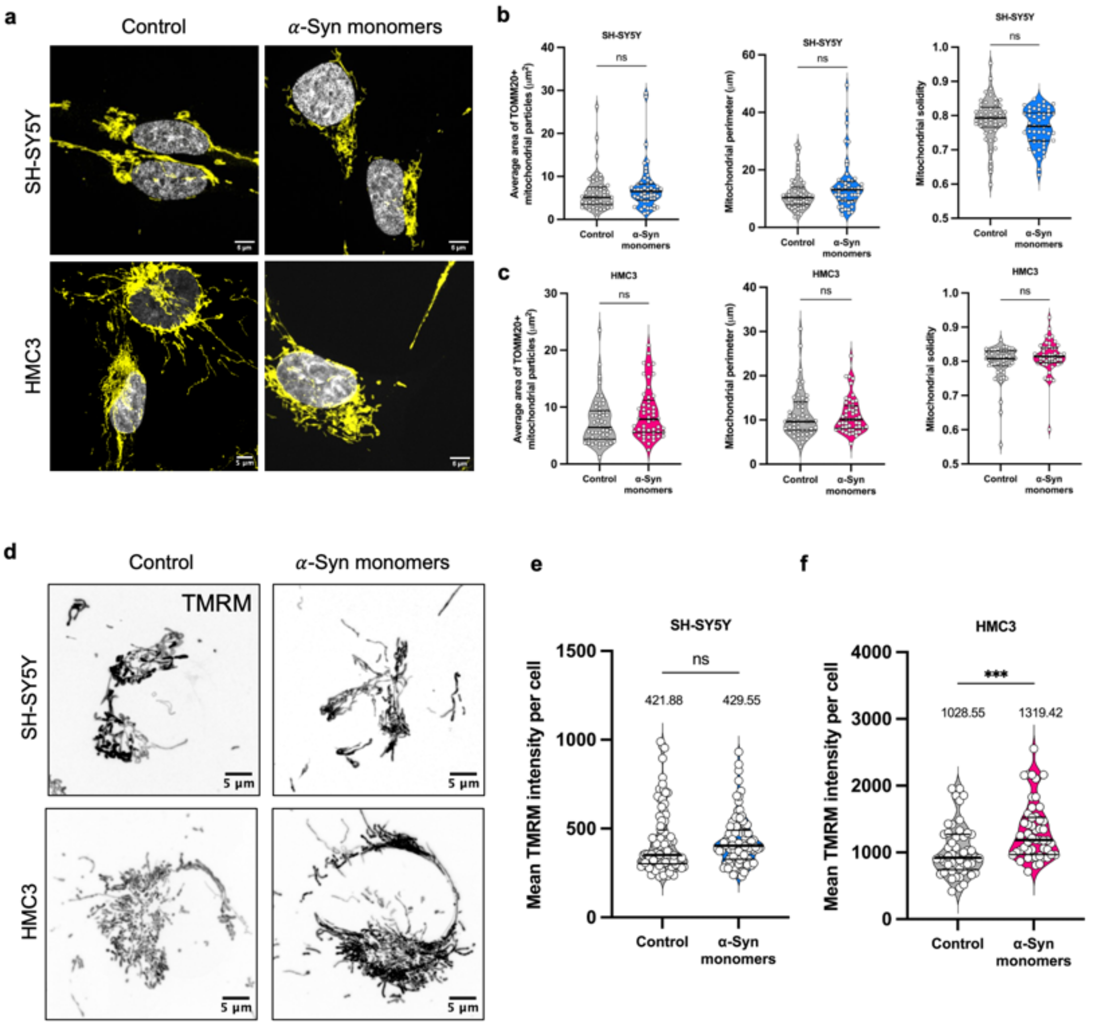

Supplementary Fig. 2. α-Syn monomers do not negatively affect mitochondria. (a) Representative confocal images depicting mitochondrial morphology in neuronal and microglial cells in control or α-Syn monomer-exposed conditions. (b-c) Quantification of mitochondrial area, perimeter and solidity in neuronal (b) and microglia (c) cells. N=3 independent experiments, n=50 cells. Statistical significance was analyzed using two-tailed Student's unpaired t-test. ns: p>0.05. Data represented as median and quartiles. (d) Representative images of TMRM stained neuronal and microglial cells treated or not with α-Syn monomers. (e-f) Quantification of mean TMRM fluorescence intensity per neuronal (e) and microglial (f) cell. N=3 independent experiments, n=100 neuronal cells per group, and n=49 control microglia and 50 α-Syn monomer-exposed microglia. Statistical significance was analyzed using two-tailed Student's unpaired t-test. ns: p>0.05; ****p=0.0006. Data represented as median and quartiles. Mean values are mentioned within the graphs.

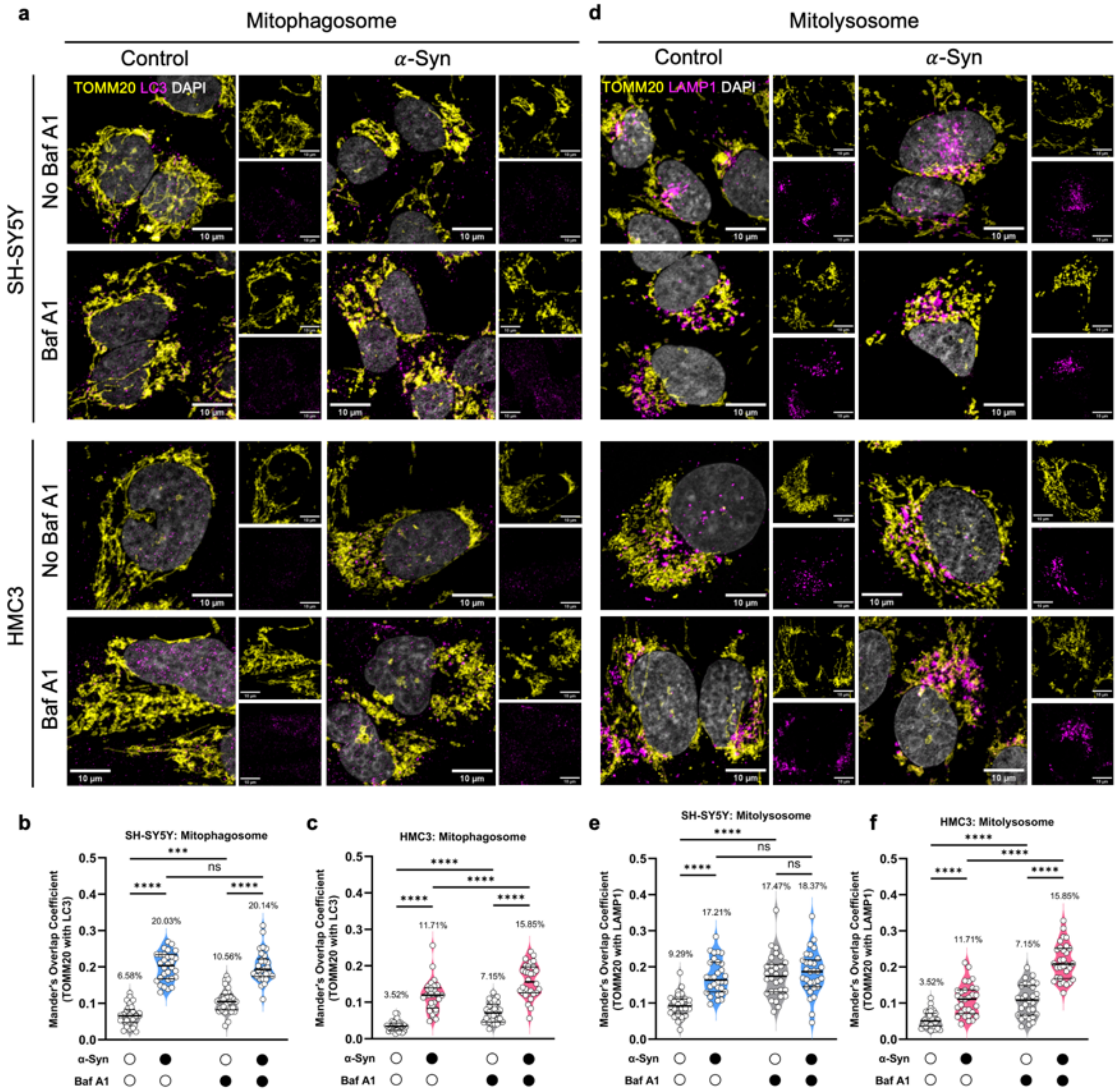

Supplementary Fig. 3. Mitophagy flux in the presence of α-Syn aggregates. (a) Representative confocal images of TOMM20+ mitochondria overlapping with LC3 (mitophagosomes) in different conditions in neuronal cells (top panels) and microglial cells (bottom panels). (b-c) Quantification of Mander's overlap coefficient of the fraction of TOMM20+ mitochondria overlapping with LC3 in neuronal cells (b) and microglial cells (c). N=3 independent experiments, n=30 cells per group. Statistical significance was analyzed using 2-Way ANOVA with Šídák's multiple comparison for (b), and uncorrected Fisher's LSD multiple comparison for (c). (d) Representative confocal images of TOMM20+ mitochondria overlapping with LAMP1 (mitolysosomes) in different conditions in neuronal cells (top panels) and microglial cells (bottom panels). (e-f) Quantification of Mander's overlap coefficient of the fraction of TOMM20+ mitochondria overlapping with

LAMP1 in neuronal cells (e) and microglial cells (f). N=3 independent experiments, n=30 cells per group. Statistical significance was analyzed using 2-Way ANOVA with Šídák's multiple comparison for (e), and uncorrected Fisher's LSD multiple comparison for (f).

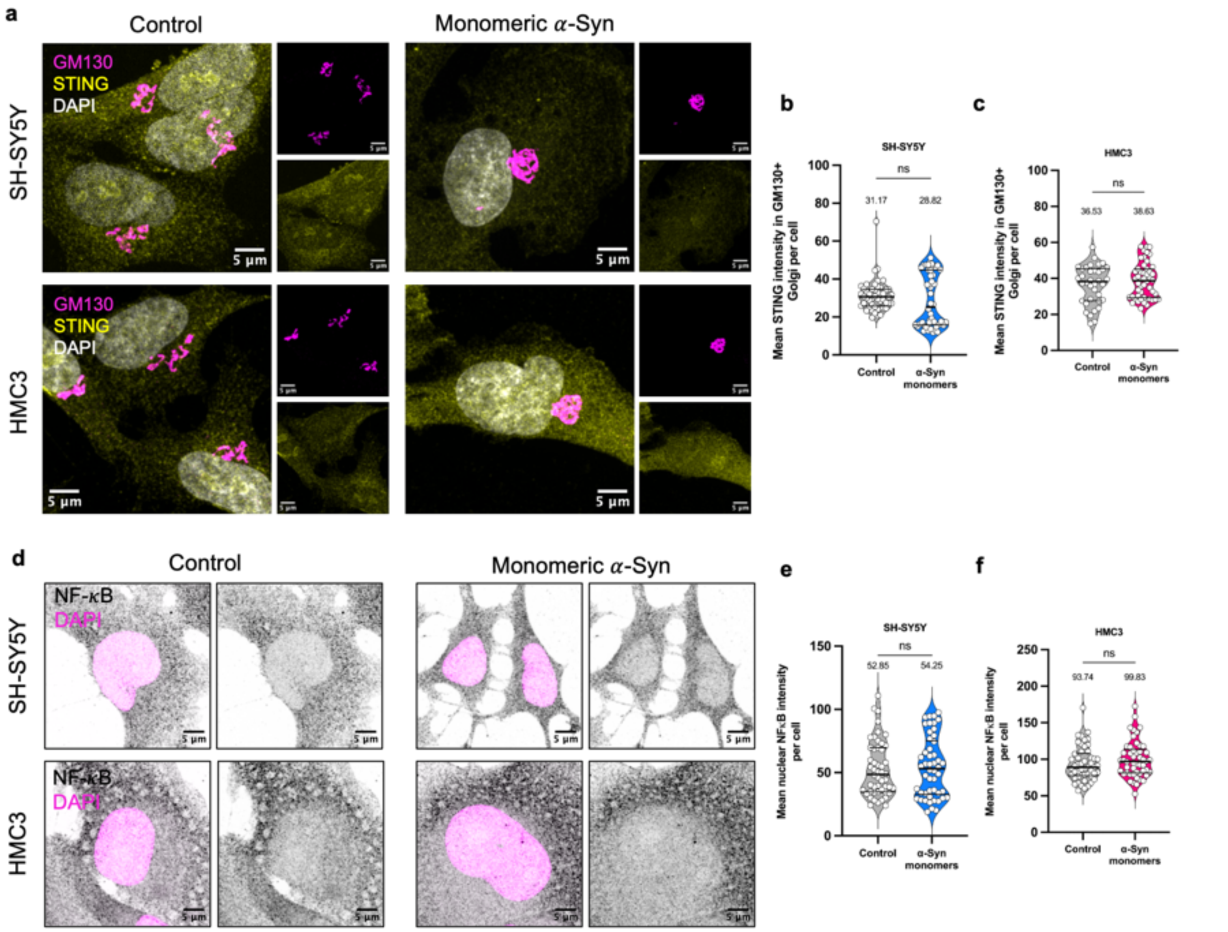

Supplementary Fig. 4. Monomeric α-Syn does not elicit an innate immune response. (a) Representative confocal images for STING translocation to GM130+ Golgi in neuronal and microglial cells treated or not with monomeric α-Syn. (b-c) Quantification of mean STING intensity in Golgi per cell. N=3 independent experiments, n=50 neuronal cells per group, and n=36 control microglia and 50 monomeric α-Syn-exposed microglia. Statistical significance was analyzed using two-tailed Student's unpaired t-test. ns: p>0.05. Data represented as median and quartiles. Mean values are mentioned within the graphs. (d) Representative confocal images for NF-κB translocation to the nucleus in neuronal and microglial cells treated or not with monomeric α-Syn. (e-f) Quantification of mean nuclear NF-κB intensty per cell. N=3 independent experiments, n=50 cells per group. Statistical significance was analyzed using two-tailed Student's unpaired t-test. ns: p>0.05. Data represented as median and quartiles. Mean values are mentioned within the graphs.

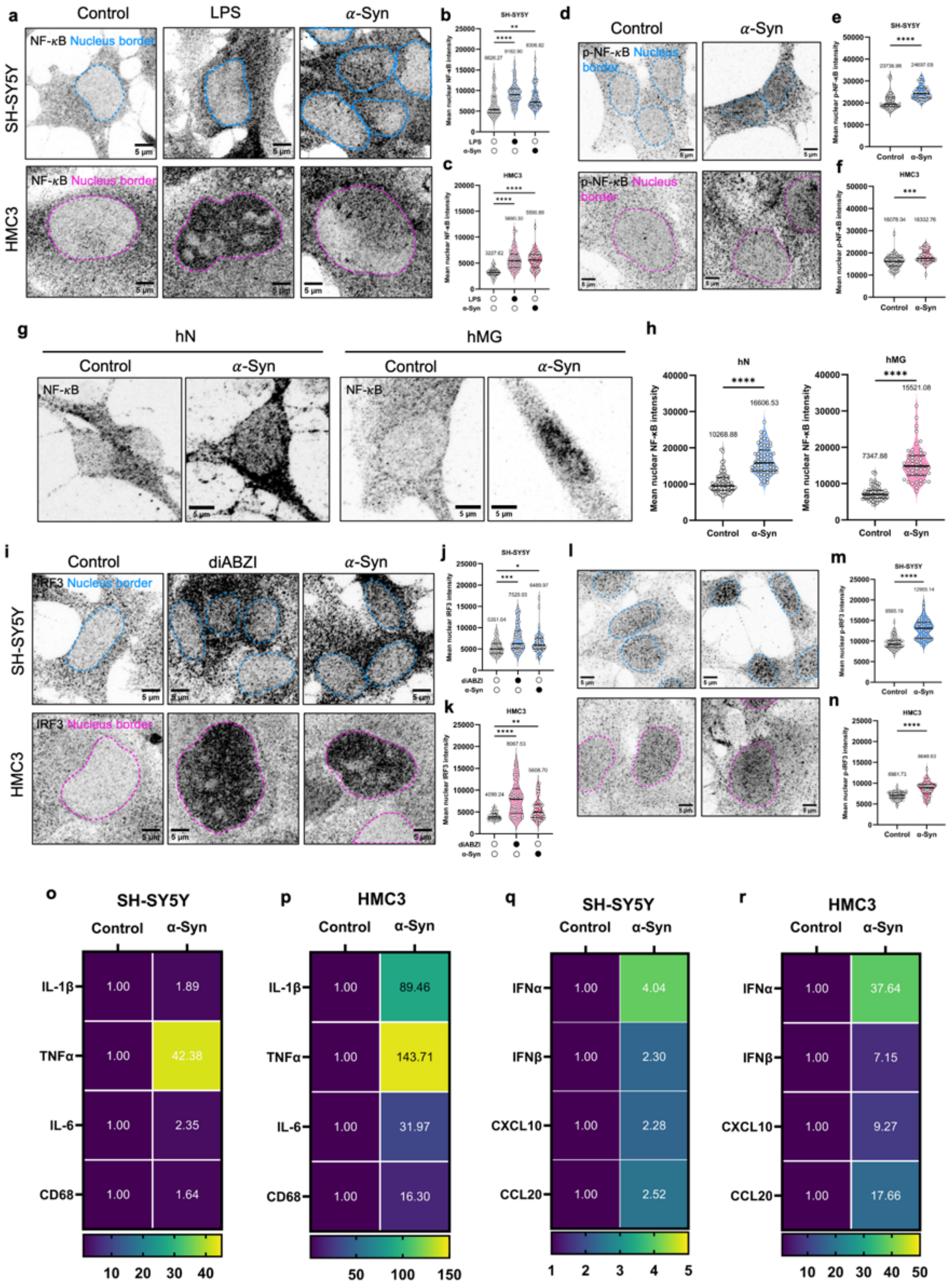

a
Control
LPS
α-Syn
SH-SY5Y
HMC3
NF-κB Nucleus border
5 μm
b
SH-SY5Y
Mean nuclear NF-κB intensity
LPS
α-Syn
c
HMC3
d
Control
α-Syn
p-NF-κB Nucleus border
e
SH-SY5Y
Mean nuclear p-NF-κB intensity
Control
α-Syn
f
HMC3
g
hN
hMG
Control
α-Syn
NF-κB
h
hN
hMG
Mean nuclear NF-κB intensity
10268.88
16606.53
7347.88
15521.08
i
Control
diABZI
α-Syn
IRF3 Nucleus border
j
SH-SY5Y
Mean nuclear IRF3 intensity
diABZI
α-Syn
k
HMC3
l
m
SH-SY5Y
Mean nuclear p-IRF3 intensity
n
HMC3
o
SH-SY5Y
Control
α-Syn
IL-1β 1.00 1.89
TNFα 1.00 42.38
IL-6 1.00 2.35
CD68 1.00 1.64
10 20 30 40
p
HMC3
Control
α-Syn
IL-1β 1.00 89.46
TNFα 1.00 143.71
IL-6 1.00 31.97
CD68 1.00 16.30
50 100 150
q
SH-SY5Y
Control
α-Syn
IFNα 1.00 4.04
IFNβ 1.00 2.30
CXCL10 1.00 2.28
CCL20 1.00 2.52
1 2 3 4 5
r
HMC3
Control
α-Syn
IFNα 1.00 37.64
IFNβ 1.00 7.15
CXCL10 1.00 9.27
CCL20 1.00 17.66
10 20 30 40 50

Supplementary Fig. 5. α-Syn aggregates induce nuclear translocation of inflammatory factors and pro-inflammatory gene expression in response to α-Syn aggregates. (a) Representative confocal images of neuronal and microglial cells assessing for nuclear levels of NF-κB. (b-c) Quantification of mean nuclear NF-κB intensity in neuronal cells (b) and microglial cells (c). N=3 independent experiments, n=50 cells per group. Statistical significance was analyzed using two-sided Kruskal-Wallis test with Dunn's multiple comparison. **p<0.01, ****p<0.0001. (d) Representative confocal images of neuronal and microglial cells assessing for nuclear levels of p-Ser-536-NF-κB. (e-f) Quantification of mean nuclear p-NF-κB intensity in neuronal cells (e) and microglial cells (f). N=3 independent experiments, n=50 cells per group. Statistical significance was analyzed using two-sided Mann-Whitney test. ***p<0.001, ****p<0.0001. (g) Representative confocal images of hiPSC-derived neurons (hN) and microglia (hMG) to assess for nuclear levels of NF-κB. (h) Quantification of mean nuclear NF-κB intensity in hNeurons and hMG. N=3 independent experiments, n=50 cells per group. Statistical significance was analyzed using two-sided Mann-Whitney test. ****p<0.0001. (i) Representative confocal images of neuronal cells (left panels) and microglial cells (right panels) to assess for nuclear levels of IRF3. (j-k) Quantification of mean nuclear IRF3 intensity in neuronal (j) and microglial (k) cells. N=3 independent experiments, n=50 cells per group. Statistical significance was analyzed using two-sided Kruskal-Wallis test with Dunn's multiple comparison. *p<0.05, ***p<0.001. (l) Representative confocal images of neuronal cells (left panels) and microglial cells (right panels) to assess for nuclear levels of p-Ser-386-IRF3. (m-n) Quantification of mean nuclear p-IRF3 intensity in neuronal cells (m) and microglial cells (n). N=3 independent experiments, n=50 cells per group. Statistical significance was analyzed using two-sided Mann-Whitney test. ****p<0.0001. Data in all violin plots are represented as median and quartiles. Mean values are mentioned within the graphs. (o-r) Heatmaps of RT-PCR-based gene expression profiles of cytokines and type I interferons in neuronal cells (o and q) and microglial cells (p and r) in response to α-Syn aggregates. N=3 independent experiments, fold changes for each gene (normalized to actin) relative to control are mentioned within the heatmaps.

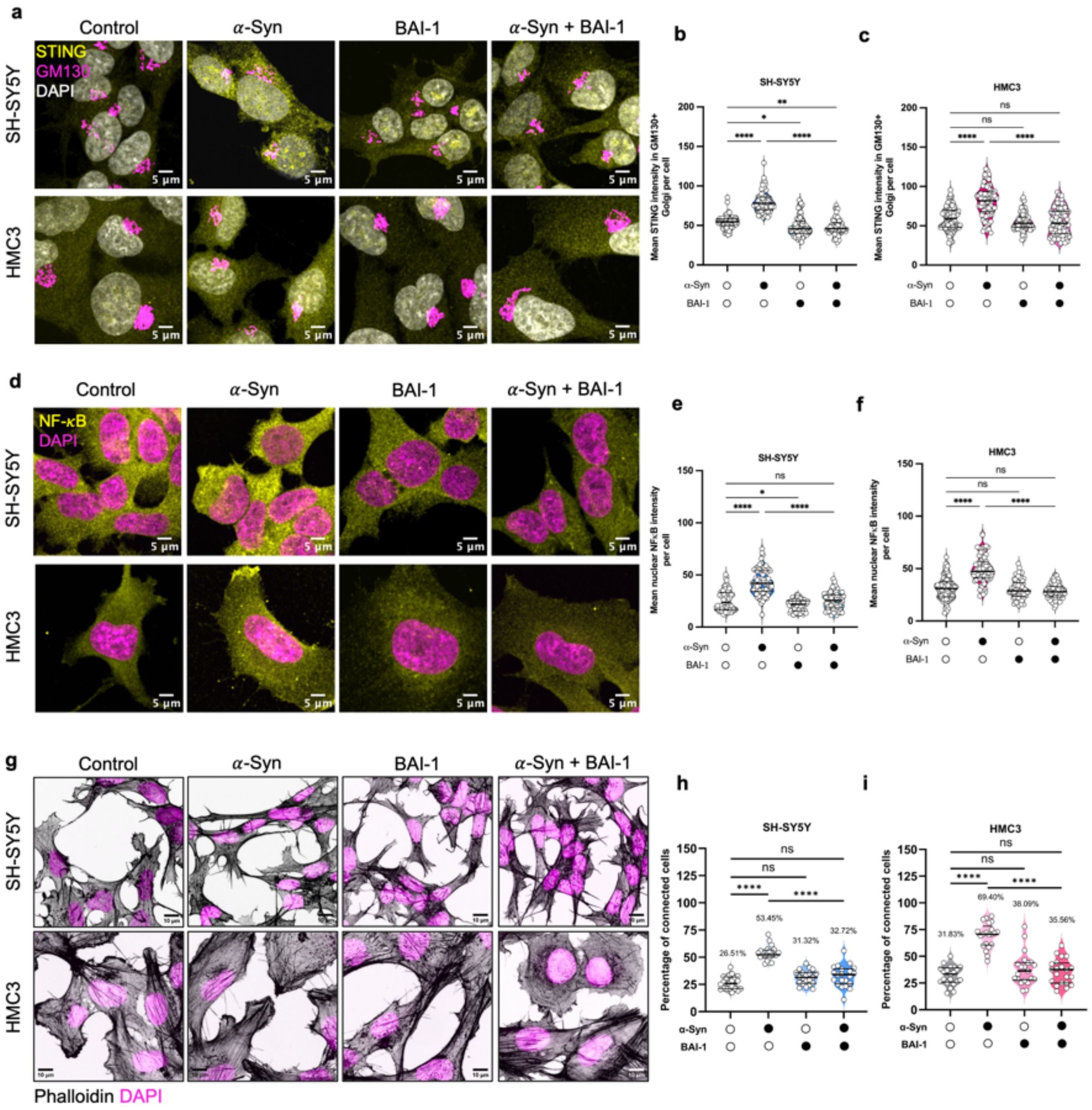

Supplementary Fig. 6. Innate immune activation and intercellular connections depend on BAX activity. (a) Representative confocal images of neuronal and microglial cells assessed for STING translocation to the Golgi upon pharmacological inhibition of BAX using BAI-1. (b-c) Quantification of mean STING fluorescence intensity on GM130+ Golgi per neuronal (b) and microglial (c) cell. N=3 independent experiments, n=75 cells per group. Statistical significance was analyzed using Kruskal Wallis test with Dunn's multiple comparison. ns: non-significant, *$p<0.05$, **$p<0.01$, ****$p<0.0001$. (d) Representative confocal images of neuronal and microglial cells assessed for NF-κB translocation to the nucleus upon pharmacological inhibition of BAX using BAI-1. (e-f) Quantification of mean NF-κB nuclear intensity per neuronal (e) and microglial (f) cell. N=3 independent experiments, n=75 cells per group. Statistical significance was analyzed

using Kruskal Wallis test with Dunn's multiple comparison. ns: non-significant, $*p<0.05$, $****p<0.0001$. (g) Representative confocal images of phalloidin-stained neuronal and microglial cells to assess intercellular connections upon pharmacological inhibition of BAX using BAI-1. (h-i) Quantification of percentage of connected cells for neuronal (h) and microglial (i) cell. N=3 independent experiments, n=20-21 fields of views per group. Statistical significance was analyzed using one-sided Brown-Forsythe and Welch One-Way ANOVA with Dunnett's T3 multiple comparison. ns: non-significant, $****p<0.0001$. Data in all graphs are represented as median and quartiles.

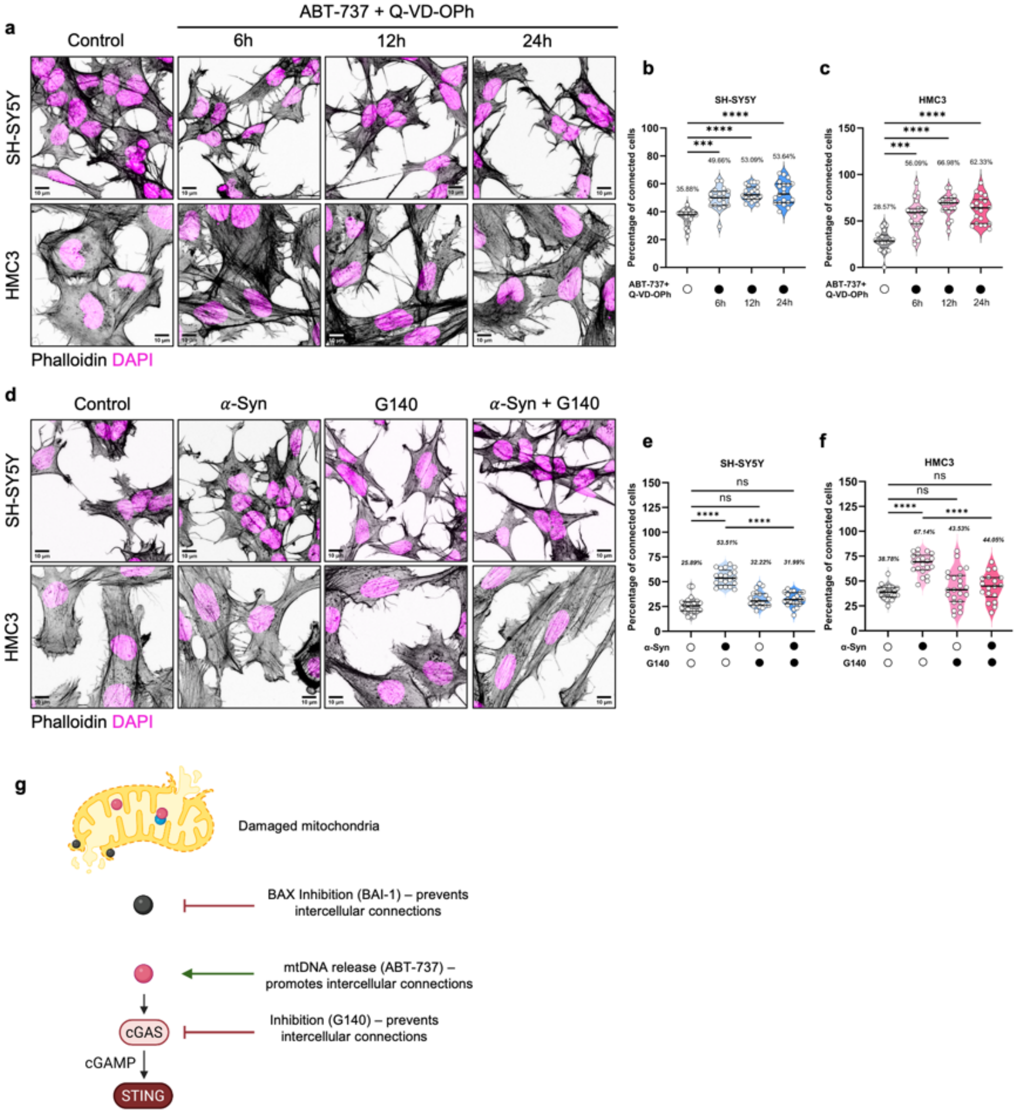

Supplementary Fig. 7. Intercellular connections as a function of mtDNA release and cGAS activity. (a) Representative phalloidin-stained images of neuronal cells (top panels) and microglial cells (bottom panels) treated with limited MOMP-inducer ABT-737 (facilitating mtDNA release) and pan-caspase inhibitor Q-VD-OPh for the designated time points. (b-c) Quantification of the percentage of connected cells in different conditions for neuronal cells (b) and microglial cells (c). N=3 independent experiments, n=18-22 fields of views for neuronal cells, and 20 fields of views for microglial cells. Statistical significance was analyzed using Kruskal-Wallis test with Dunn's multiple comparison. ***p<0.001, ****p<0.0001. (d) Representative phalloidin-stained images of neuronal cells (top panels) and microglial cells (bottom panels) treated with α-Syn alone, the cGAS inhibitor G140 alone, or co-treated with both. (e-f) Quantification of the percentage of connected cells in different conditions for neuronal cells (e) and microglial cells (f). N=3 independent experiments, n=21 fields of views for neuronal cells, and 18-23 fields of

views for microglial cells. Statistical significance was analyzed using Brown-Forsythe and Welch ANOVA with Dunnett's T3 multiple comparison. ns: $p>0.05$, ****$p<0.0001$. Data represented as median and quartiles, with mean percentages of connections mentioned within the graphs. (g) Schematic representation of mtDNA release and cGAS as positive regulators of intercellular connections. Created in BioRender. MAYA, S. (2026) https://BioRender.com/gn345yb.

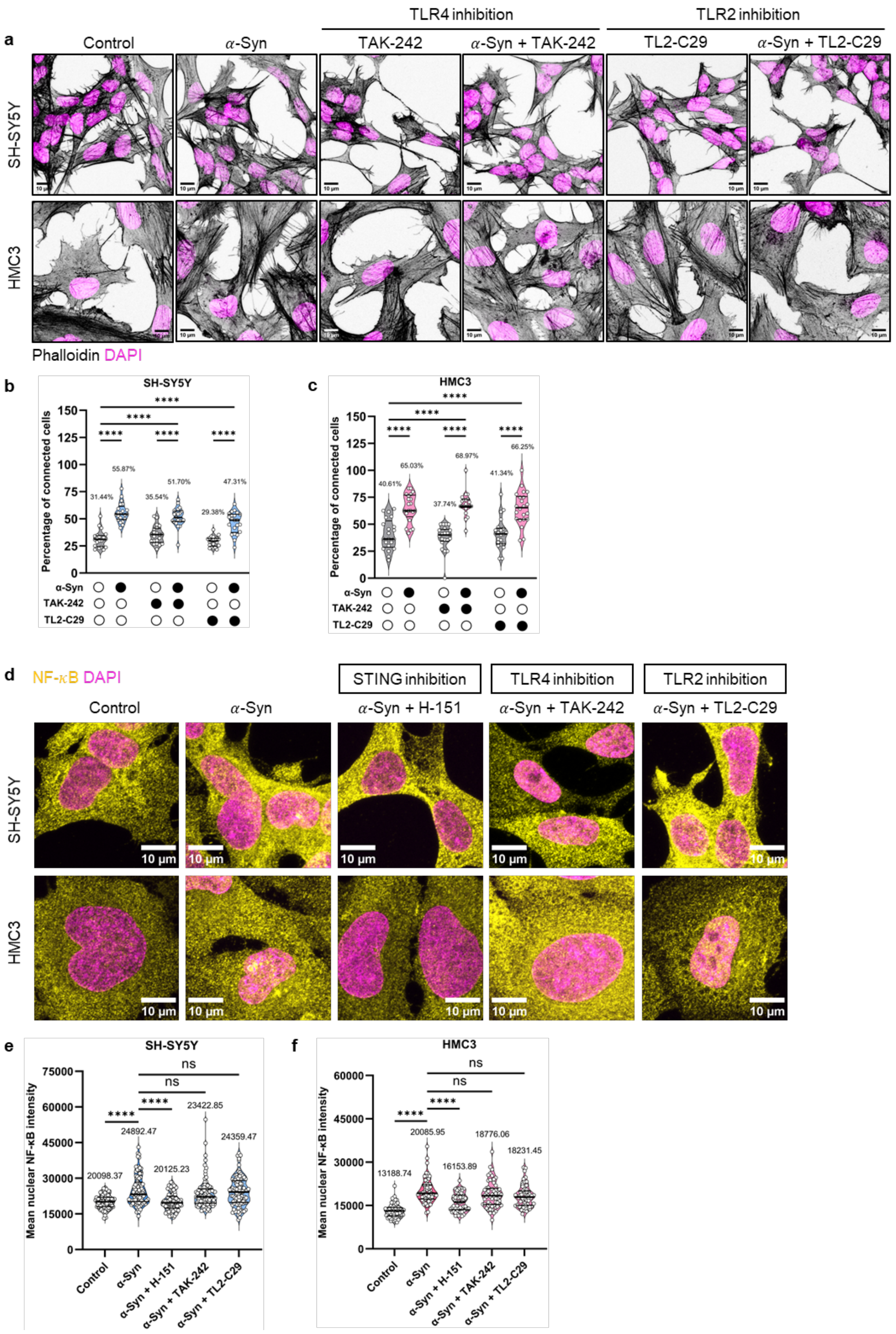
a
TLR4 inhibition
TLR2 inhibition
Control
α-Syn
TAK-242
α-Syn + TAK-242
TL2-C29
α-Syn + TL2-C29
SH-SY5Y
HMC3
Phalloidin DAPI
b
SH-SY5Y
Percentage of connected cells
31.44%
55.87%
35.54%
51.70%
29.38%
47.31%
α-Syn
TAK-242
TL2-C29
c
HMC3
40.61%
65.03%
37.74%
68.97%
41.34%
66.25%
d
NF-κB DAPI
STING inhibition
TLR4 inhibition
TLR2 inhibition
Control
α-Syn
α-Syn + H-151
α-Syn + TAK-242
α-Syn + TL2-C29
10 µm
e
SH-SY5Y
Mean nuclear NF-κB intensity
ns
20098.37
24892.47
20125.23
23422.85
24359.47
Control
α-Syn
α-Syn + H-151
α-Syn + TAK-242
α-Syn + TL2-C29
f
HMC3
13188.74
20085.95
16153.89
18776.06
18231.45

Supplementary Fig. 8. Intercellular connections and NF-κB translocation to the nucleus in response to α-Syn after 16h of aggregate exposure is independent of TLR2/4. (a) Representative phalloidin-stained images of neuronal and microglial cells in different conditions. (b-c) Quantification of the percentage of connected cells for neuronal cells (b) and microglial cells (c). N=3 independent experiments, n=20-22 fields of views for neuronal cells, and 21-22 fields of views for microglial cells. Statistical significance was analyzed using Two-Way ANOVA with Tukey's multiple comparison. ****p<0.0001. (d) Representative confocal images of neuronal and microglial cells to assess for nuclear translocation of NF-κB upon α-Syn exposure alone, or together with STING, TLR4, and TLR2 inhibitors. (e-f) Quantification of mean nuclear NF-κB intensity in different conditions for neuronal cells (e) and microglial cells (f). N=3 independent experiments, n=100 cells per group for (e) and 60 cells per group for (f). Statistical significance was analyzed using Kruskal-Wallis test with Dunn's multiple comparison. ns: p>0.05, ****p<0.0001. Data represented as median and quartiles, mean values mentioned within the graphs.

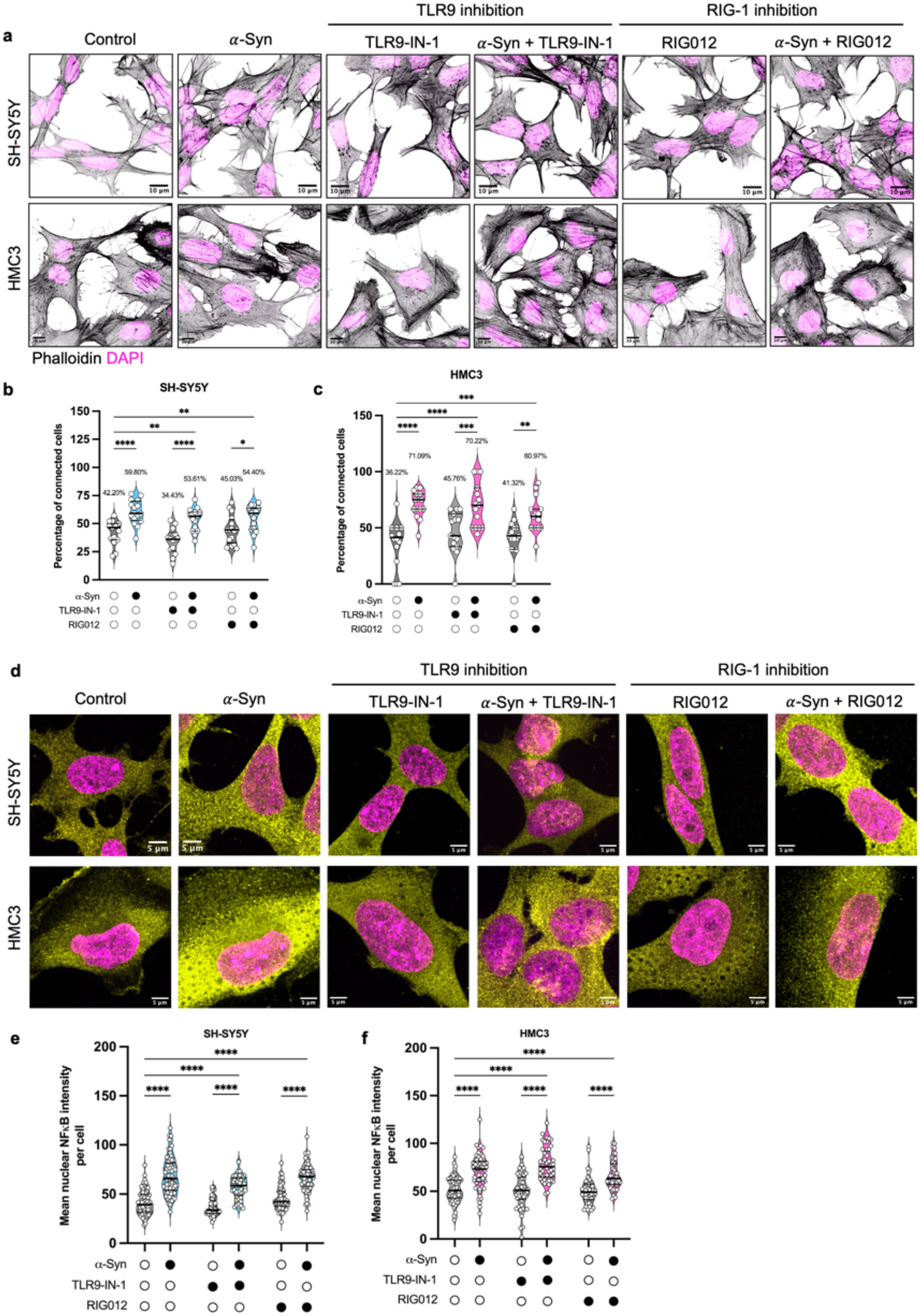

a
TLR9 inhibition
RIG-1 inhibition
Control
α-Syn
TLR9-IN-1
α-Syn + TLR9-IN-1
RIG012
α-Syn + RIG012
SH-SY5Y
HMC3
Phalloidin DAPI
b
SH-SY5Y
Percentage of connected cells
42.20%
59.80%
34.43%
53.61%
45.03%
54.40%
α-Syn
TLR9-IN-1
RIG012
c
HMC3
Percentage of connected cells
36.22%
71.09%
45.76%
70.22%
41.32%
60.97%
α-Syn
TLR9-IN-1
RIG012
d
TLR9 inhibition
RIG-1 inhibition
Control
α-Syn
TLR9-IN-1
α-Syn + TLR9-IN-1
RIG012
α-Syn + RIG012
SH-SY5Y
HMC3
e
SH-SY5Y
Mean nuclear NFκB intensity per cell
α-Syn
TLR9-IN-1
RIG012
f
HMC3
Mean nuclear NFκB intensity per cell
α-Syn
TLR9-IN-1
RIG012

Supplementary Fig. 9. Intercellular connections and NF-κB translocation to the nucleus in response to α-Syn after 16h of aggregate exposure is independent of TLR9 and RIG-1. (a) Representative phalloidin-stained images of neuronal and microglial cells in different conditions. (b-c) Quantification of the percentage of connected cells for neuronal cells (b) and microglial cells (c). N=3 independent experiments, n=14-16 fields of views for neuronal cells, and 15-16 fields of views for microglial cells. Statistical significance was analyzed using Two-Way ANOVA with Uncorrected Fisher's LSD. *p<0.05, **p<0.01, ***p<0.001, ****p<0.0001. (d) Representative confocal images of neuronal and microglial cells to assess for nuclear translocation of NF-κB upon α-Syn exposure alone, or together with STING, TLR4, and TLR2 inhibitors. (e-f) Quantification of mean nuclear NF-κB intensity in different conditions for neuronal cells (e) and microglial cells (f). N=2 independent experiments, n=67-75 neuronal cells and 59-60 microglial cells. Statistical significance was analyzed using Two-Way ANOVA with Uncorrected Fisher's LSD. ****p<0.0001. Data represented as median and quartiles.

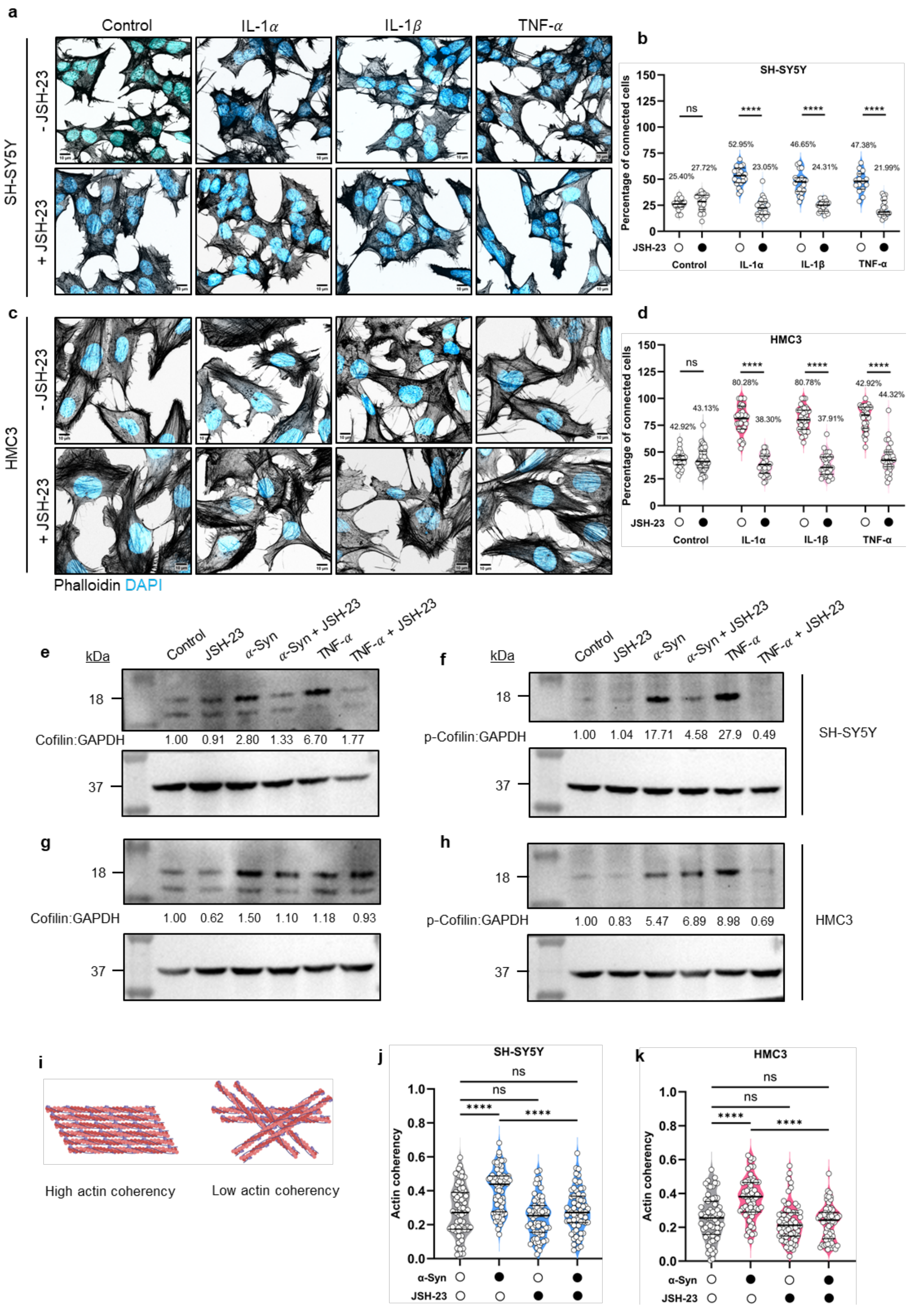

a
Control
IL-1α
IL-1β
TNF-α
SH-SY5Y
- JSH-23
+ JSH-23
b
SH-SY5Y
Percentage of connected cells
ns
****
25.40%
27.72%
52.95%
23.05%
46.65%
24.31%
47.38%
21.99%
JSH-23
Control
IL-1α
IL-1β
TNF-α
c
HMC3
- JSH-23
+ JSH-23
Phalloidin DAPI
d
HMC3
Percentage of connected cells
42.92%
43.13%
80.28%
38.30%
80.78%
37.91%
42.92%
44.32%
e
kDa
Control
JSH-23
α-Syn
α-Syn + JSH-23
TNF-α
TNF-α + JSH-23
18
Cofilin:GAPDH 1.00 0.91 2.80 1.33 6.70 1.77
37
f
p-Cofilin:GAPDH 1.00 1.04 17.71 4.58 27.9 0.49
SH-SY5Y
g
Cofilin:GAPDH 1.00 0.62 1.50 1.10 1.18 0.93
h
p-Cofilin:GAPDH 1.00 0.83 5.47 6.89 8.98 0.69
HMC3
i
High actin coherency
Low actin coherency
j
SH-SY5Y
Actin coherency
α-Syn
JSH-23
k
HMC3

Supplementary Fig. 10. Inflammation-induced intercellular connections are NF-κB dependent, caused by changes in actin organization and Cofilin activity. (a) Representative phalloidin-stained images of neuronal cells treated with pro-inflammatory cytokines in the absence (top panels) or presence (bottom panels) of the NF-κB inhibitor JSH-23. (b) Quantification of the percentage of connected neuronal cells in different conditions. (c) Representative phalloidin-stained images of microglial cells treated with pro-inflammatory cytokines in the absence (top panels) or presence (bottom panels) of the NF-κB inhibitor JSH-23. (b) Quantification of the percentage of connected microglial cells in different conditions. For both (b) and (d): N=3 independent experiments, n=15-21 fields of views for neuronal cells and 23-38 fields of views for microglial cells. Statistical significance was analyzed using Two-Way ANOVA with Tukey's multiple comparison. ns: $p>0.05$, ****$p<0.0001$. Data represented as median and quartiles, with mean percentages mentioned within the graphs. (e-f) Representative western blots for Cofilin (e) and p-Ser3-Cofilin (f) in different treatment conditions for neuronal cells. N=2 independent experiments. Mean fold difference (normalized to GAPDH) relative to control is mentioned. (g-h) Representative western blots for Cofilin (g) and p-Ser3-Cofilin (h) in different treatment conditions for microglial cells. N=2 independent experiments. Mean fold difference (normalized to GAPDH) relative to control is mentioned. (i) Schematic representation of actin filament organization depicting high or low coherency. (j-k) Quantification of actin coherency for neuronal cells (j) and microglial cells (k) in control conditions, or treated with α-Syn or JSH-23 alone, or co-treated with both. N=3 independent experiments, n=64-69 neuronal cells, and 63-66 microglial cells. Statistical significance was analyzed using Brown-Forsythe and Welch ANOVA with Dunnett's T3 multiple comparison. ns: $p>0.05$, ****$p<0.0001$. Data represented as median and quartiles. Schematic in panel (i) was Created in BioRender. MAYA, S. (2026) https://BioRender.com/biyqi6d.

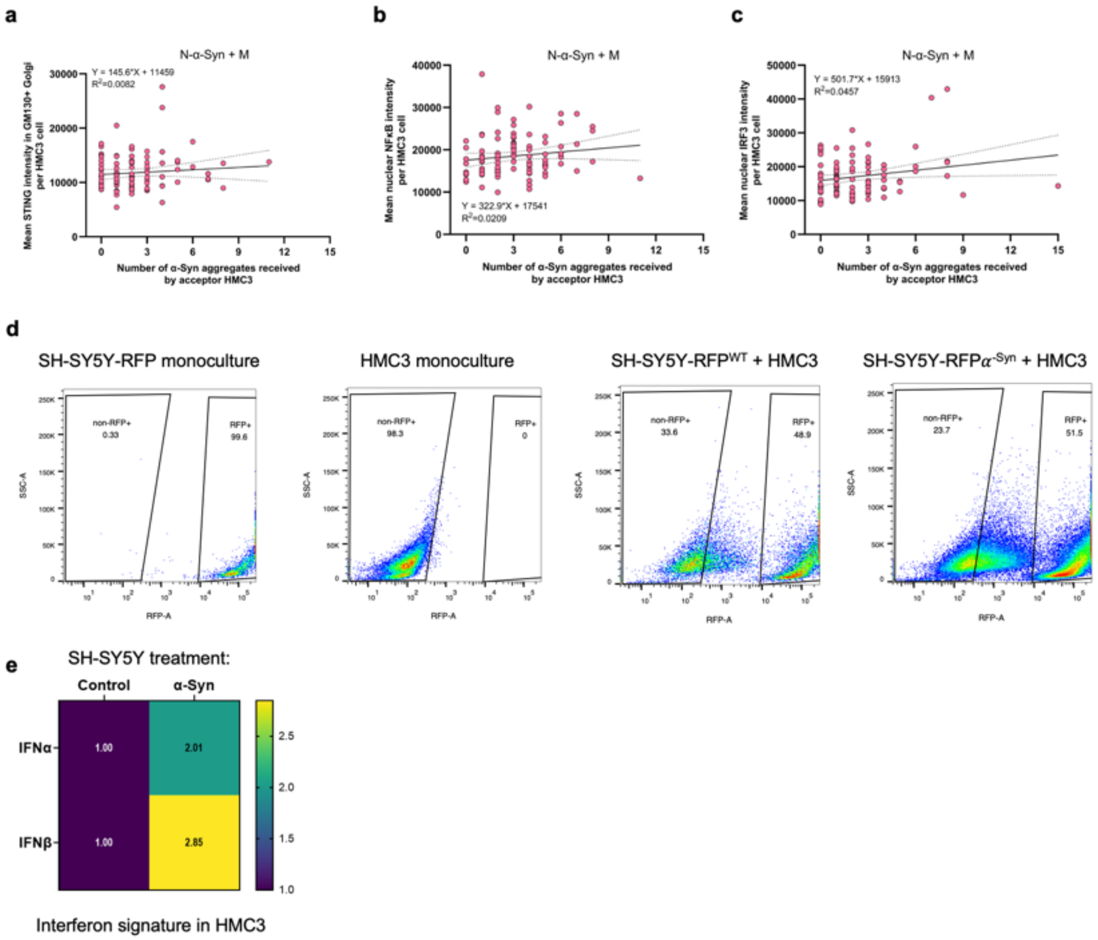

Supplementary Fig. 11. **Bystander inflammation in microglia is not correlated to the number of α-Syn aggregates they receive from neuronal cells, and microglial interferon response**. (a-c) Correlation graphs of the number of α-Syn aggregates received per cell, and STING translocation to GM130+ Golgi (a), NF-κB translocation to the nucleus (b) and IRF3 translocation to the nucleus (c) in those very cells. Solid lines represent simple linear regression, and 95% confidence intervals are indicated by the dashed lines. (d) Gating strategy to isolate RFP+ and RFP- cells from SH-RFP and HMC3 co-cultures. (e) Microglia isolated from co-cultures with naïve (control) or α-Syn-burdened neuronal cells after flow cytometry-based sorting were analyzed for type I interferon responses.

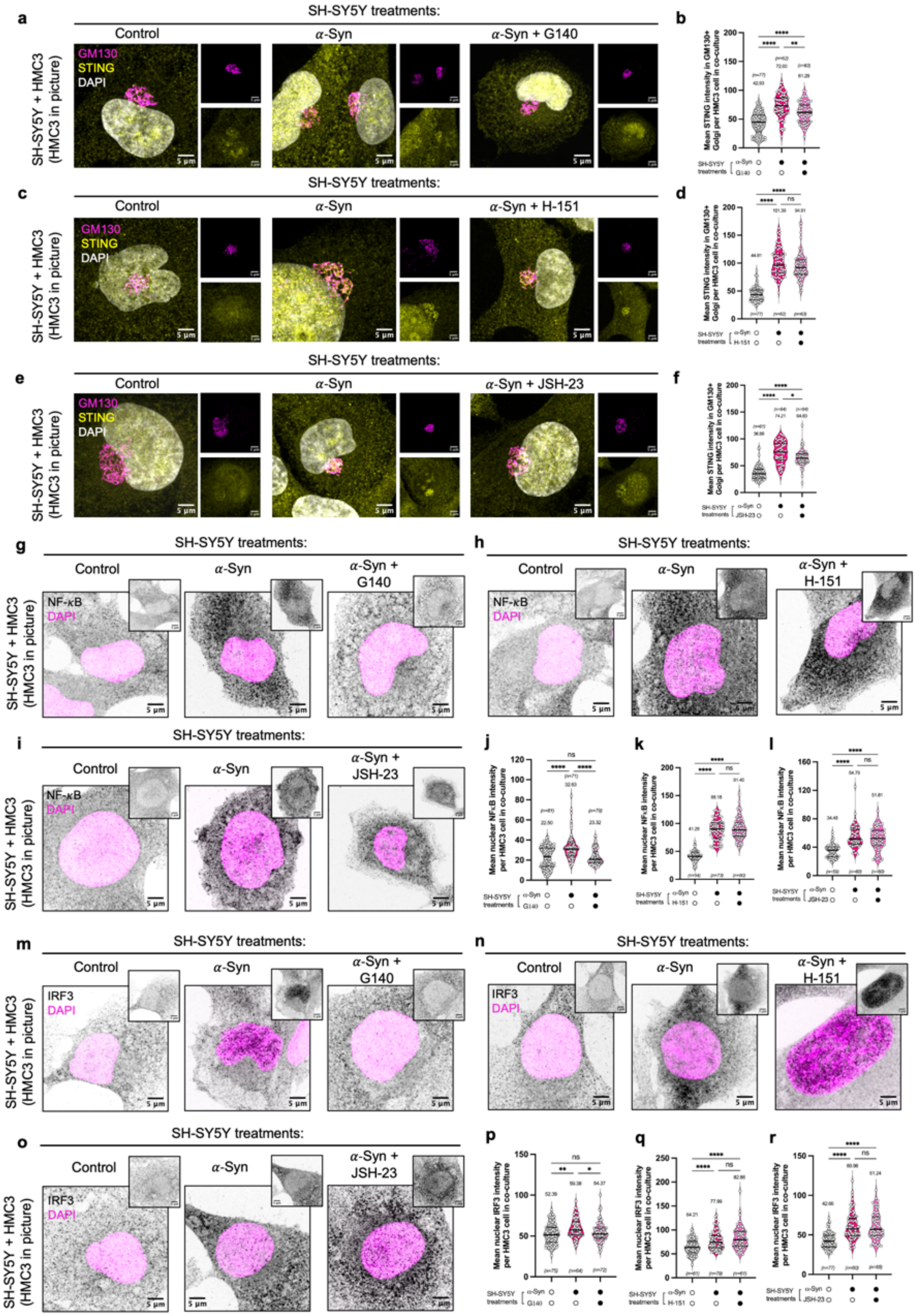

SH-SY5Y treatments:
Control
α-Syn
α-Syn + G140
α-Syn + H-151
α-Syn + JSH-23
GM130
STING
DAPI
NF-κB
IRF3
SH-SY5Y + HMC3 (HMC3 in picture)
5 μm
Mean STING intensity in GM130+ Golgi per HMC3 cell in co-culture
Mean nuclear NF-κB intensity per HMC3 cell in co-culture
Mean nuclear IRF3 intensity per HMC3 cell in co-culture
SH-SY5Y treatments
ns

Supplementary Fig. 12. Bystander inflammation in microglia is neuronal cGAS-dependent. Bystander inflammatory responses in microglia were assessed by co-culturing naïve, α-Syn treated, or α-Syn and cGAS/STING/NF-κB inhibitor treated neuronal cells with microglia. (a) Representative confocal images assessing the role of neuronal cGAS in microglial STING activation. (b) Quantification of mean STING intensity on GM130+ Golgi. N=2 independent experiments, n=62-77 cells. Statistical significance was analyzed using Brown-Forsythe and Welch ANOVA with Games-Howell's multiple comparison. **p<0.01, ***p<0.0001. (c) Representative confocal images assessing the role of neuronal STING in microglial STING activation. (d) Quantification of mean STING intensity on GM130+ Golgi. N=2 independent experiments, n=71-79 cells. Statistical significance was analyzed using Brown-Forsythe and Welch ANOVA with Games-Howell's multiple comparison. ns: non-significant, p>0.05, ****p<0.0001. (e) Representative confocal images assessing the role of neuronal NF-κB in microglial STING activation. (f) Quantification of mean STING intensity on GM130+ Golgi. N=2 independent experiments, n=61-64 cells. Statistical significance was analyzed using Brown-Forsythe and Welch ANOVA with Games-Howell's multiple comparison. ns: non-significant, p>0.05, ***p<0.0001. (g-i) Same as (a-f) but assessing for nuclear occupancy of NF-κB per microglial cell. (j-l) Corresponding quantifications of mean nuclear NF-κB intensity in microglia upon neuronal cGAS (j), STING (k) and NF-κB (l) inhibitions. N=2 independent experiments, n=71-81 cells for (j), 54-80 cells for (k) and 59-60 cells for (l). Statistical significance was analyzed using Brown-Forsythe and Welch ANOVA with Games-Howell's multiple comparison. ns: non-significant, p>0.05, ****p<0.0001. (m-o) Same as previous but assessing for nuclear occupancy of IRF3 per microglial cell. (p-r) Corresponding quantifications of mean nuclear IRF3 intensity in microglia upon neuronal cGAS (p), STING (q) and NF-κB (r) inhibitions. N=2 independent experiments, n=64-75 cells for (p), 61-79 cells for (q) and 65-80 cells for (r). Statistical significance was analyzed using Brown-Forsythe and Welch ANOVA with Games-Howell's multiple comparison. ns: non-significant, p>0.05, ****p<0.0001. Data represented as median and quartiles.

Raw uncropped blot images for Figure S10e-h

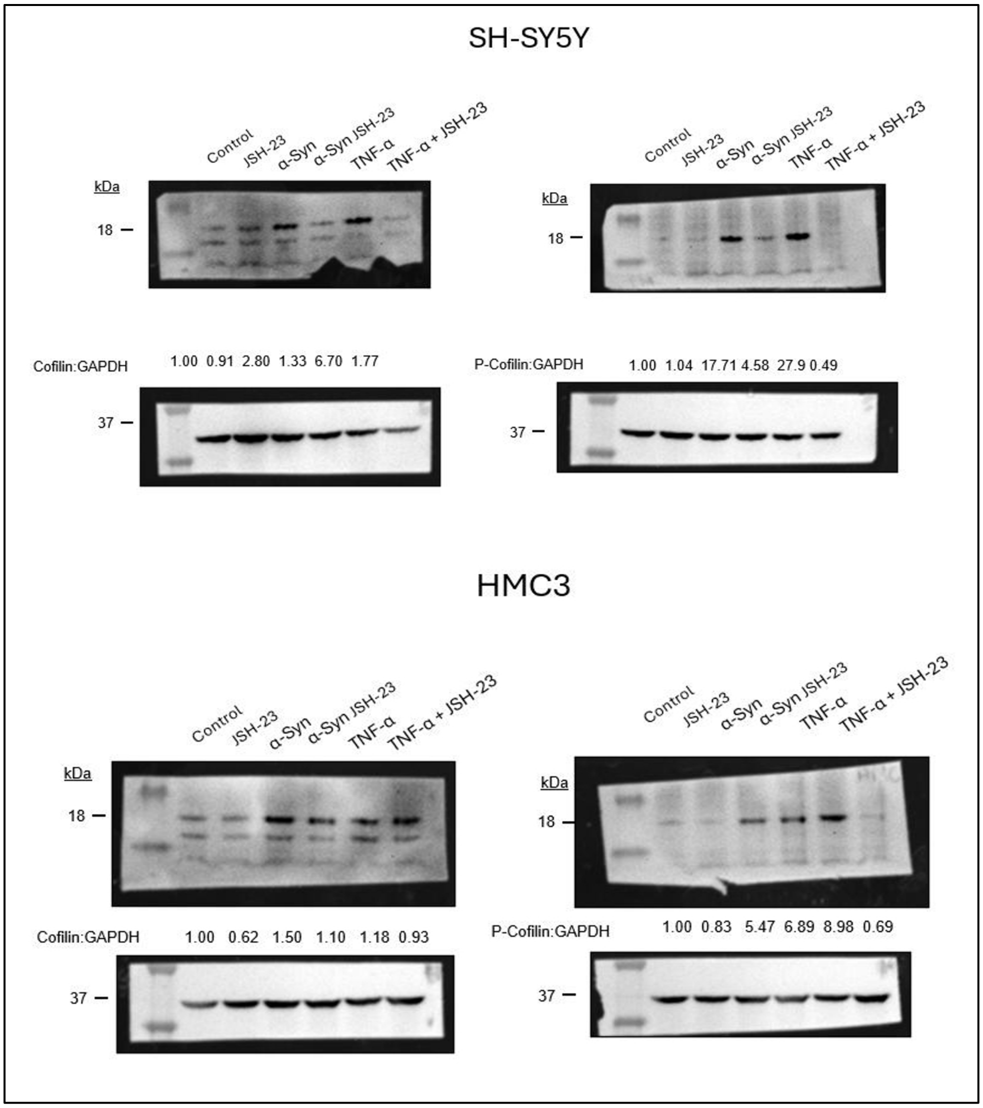

Supplement: Supplementary file 1 — Supplementary Information [file 41467_2026_73136_MOESM1_ESM.pdf]
