# Supplementary material for: α-Synuclein aggregates induce mitochondrial damage and trigger innate immunity to drive neuron–microglia communication
Source: Nat Commun. 2026 May 15;17:6462. doi: 10.1038/s41467-026-73136-7 (PMC13376753; doi:10.1038/s41467-026-73136-7)
Supplement: Supplementary file 2 — Reporting Summary [file 41467_2026_73136_MOESM2_ESM.pdf]

nature portfolio

Corresponding author(s): Chiara Zurzolo
Last updated by author(s): Apr 27, 2026

Reporting Summary

Nature Portfolio wishes to improve the reproducibility of the work that we publish. This form provides structure for consistency and transparency in reporting. For further information on Nature Portfolio policies, see our Editorial Policies and the Editorial Policy Checklist.

Statistics

For all statistical analyses, confirm that the following items are present in the figure legend, table legend, main text, or Methods section.

n/a	Confirmed	
☐	☒	The exact sample size (*n*) for each experimental group/condition, given as a discrete number and unit of measurement
☐	☒	A statement on whether measurements were taken from distinct samples or whether the same sample was measured repeatedly
☐	☒	The statistical test(s) used AND whether they are one- or two-sided<br>*Only common tests should be described solely by name; describe more complex techniques in the Methods section.*
☒	☐	A description of all covariates tested
☐	☒	A description of any assumptions or corrections, such as tests of normality and adjustment for multiple comparisons
☐	☒	A full description of the statistical parameters including central tendency (e.g. means) or other basic estimates (e.g. regression coefficient) AND variation (e.g. standard deviation) or associated estimates of uncertainty (e.g. confidence intervals)
☐	☒	For null hypothesis testing, the test statistic (e.g. F, t, r) with confidence intervals, effect sizes, degrees of freedom and P value noted<br>*Give P values as exact values whenever suitable.*
☒	☐	For Bayesian analysis, information on the choice of priors and Markov chain Monte Carlo settings
☒	☐	For hierarchical and complex designs, identification of the appropriate level for tests and full reporting of outcomes
☒	☐	Estimates of effect sizes (e.g. Cohen's d, Pearson's r), indicating how they were calculated

Our web collection on statistics for biologists contains articles on many of the points above.

Software and code

Policy information about availability of computer code

Data collection	Fluorescent Images were acquired using Zen Blue (LSM confocal), Zen Black (Elyra 7 SM) softwares of Zeiss, or Nikon NIS-Elements software of Nikon Eclipse Ti2 spinning disk confocal microscope.
Data analysis	Image analyses was performed using FIJI. Statistical analyses were performed and graphs were prepared in GraphPad Prism 10.0.

For manuscripts utilizing custom algorithms or software that are central to the research but not yet described in published literature, software must be made available to editors and reviewers. We strongly encourage code deposition in a community repository (e.g. GitHub). See the Nature Portfolio guidelines for submitting code & software for further information.

Data

Policy information about availability of data

All manuscripts must include a data availability statement. This statement should provide the following information, where applicable:

- Accession codes, unique identifiers, or web links for publicly available datasets
- A description of any restrictions on data availability
- For clinical datasets or third party data, please ensure that the statement adheres to our policy

All data are presented as main figures, or supplementary figures.

Research involving human participants, their data, or biological material

Policy information about studies with human participants or human data. See also policy information about sex, gender (identity/presentation), and sexual orientation and race, ethnicity and racism.

Reporting on sex and gender	N/A
Reporting on race, ethnicity, or other socially relevant groupings	N/A
Population characteristics	N/A
Recruitment	N/A
Ethics oversight	N/A

Note that full information on the approval of the study protocol must also be provided in the manuscript.

Field-specific reporting

Please select the one below that is the best fit for your research. If you are not sure, read the appropriate sections before making your selection.

[x] Life sciences [ ] Behavioural & social sciences [ ] Ecological, evolutionary & environmental sciences

For a reference copy of the document with all sections, see nature.com/documents/nr-reporting-summary-flat.pdf

Life sciences study design

All studies must disclose on these points even when the disclosure is negative.

Sample size	No statistical method was used to predetermine sample size. Sample size was determined empirically and was based on previous experience from similar studies. In vitro experiments were performed including at least 3 biological replicates to confirm reproducibility. Sample or group sizes of the experiments were chosen based on previous experience and published studies.
Data exclusions	No data points/biological replicates were excluded from analyses.
Replication	All experiments were repeated atleast 3 times, unless otherwise stated, with independent biological replicates.
Randomization	Treatment groups were designated randomly between wells and plates.
Blinding	Investigators were not blinded during experiments and analyses. There were defined groups and blinding was not necessary

Reporting for specific materials, systems and methods

We require information from authors about some types of materials, experimental systems and methods used in many studies. Here, indicate whether each material, system or method listed is relevant to your study. If you are not sure if a list item applies to your research, read the appropriate section before selecting a response.

Materials & experimental systems

n/a	Involved in the study	
[ ]	[x]	Antibodies
[ ]	[x]	Eukaryotic cell lines
[x]	[ ]	Palaeontology and archaeology
[x]	[ ]	Animals and other organisms
[x]	[ ]	Clinical data
[x]	[ ]	Dual use research of concern
[x]	[ ]	Plants

Methods

n/a	Involved in the study	
[x]	[ ]	ChIP-seq
[ ]	[x]	Flow cytometry
[x]	[ ]	MRI-based neuroimaging

Antibodies

Antibodies used	TOMM20 (Santa Cruz, sc-17764; 1:400), Cytochrome c (BD Pharmigen, 556432, 1:400), dsDNA (Progen, clone AC-30-10, 690014; 1:100), TFAM (Invitrogen, PA5-29571; 1:400), GM130 (BD Biosciences, 610823; 1:100), STING (Invitrogen, PA5-23381; 1:100), phospho-Ser172-TBK1 (Cell Signaling Technology, D52C2; 1:50), NF-κB (Thermo Fischer, 51-0500; 1:100), phospho-Ser536-NF-κB (Invitrogen, Ma5-15160, T.849.2; 1:100), IRF3 (Cell Signaling Technology, D6I4C; 1:100), phospho-Ser386-IRF3 (Cell Signaling Technology, E7J8G; 1:100), activated BAX-6A7 (Santa Cruz, sc23959; 1:100; kind gift from Julien Prudent), LC3B (Cell Signaling Technology, D11, 1:500), LAMP1 (DSHB, H4A3, 1:100), Cofilin (Invitrogen, PA5-17372; 1:1000 for western blotting), phospho-Ser3-

	Cofilin (Invitrogen, PA5-17752; 1:1000 for western blotting), GAPDH (Sigma, G9545; 1:5000 for western blotting).
Validation	All antibodies used were commercially available, validated, and cited by various studies. Webpage links, within square brackets, for the listed antibodies with confirmed species reactivity are mentioned below:<br><br>TOMM20 (Santa Cruz, sc-17764) : Confirmed species reactivity- mouse, rat and human [https://www.scbt.com/p/tom20-antibody-f-10?srsltid=AfmBOorJFfb1Wf6ujDFfgj9cfF6_4DjpYMJo2i5RB-bBbg4stceMo1tv]<br>Cytochrome c (BD Pharmigen, 556432) : Confirmed species reactivity- Human (QC Testing), Mouse, Rat (Reactivity Confirmed in Development) [https://www.bdbiosciences.com/en-us/products/reagents/microscopy-imaging-reagents/immunofluorescence-reagents/purified-mouse-anti-cytochrome-c.556432?tab=product_details]<br>dsDNA (Progen, clone AC-30-10, 690014) : Confirmed species reactivity- All species [https://us.progen.com/anti-dna-mouse-monoclonal-ac-30-10-liquid-purified/690014]<br>TFAM (Invitrogen, PA5-29571) : Confirmed species reactivity- Human, Mouse, Rat [https://www.thermofisher.com/antibody/product/TFAM-Antibody-Polyclonal/PA5-29571]<br>GM130 (BD Biosciences, 610823) : Confirmed species reactivity- Rat (QC Testing), Human, Mouse, Dog (Tested in Development) [https://www.bdbiosciences.com/en-us/products/reagents/microscopy-imaging-reagents/immunofluorescence-reagents/purified-mouse-anti-gm130.610823?tab=product_details]<br>STING (Invitrogen, PA5-23381) : Confirmed species reactivity- Human, Mouse, Non-human primate, Rhesus monkey [https://www.thermofisher.com/antibody/product/STING-Antibody-Polyclonal/PA5-23381]<br>phospho-Ser172-TBK1 (Cell Signaling Technology, D52C2) : Confirmed species reactivity- Human, Mouse, Rat [https://www.cellsignal.com/products/primary-antibodies/phospho-tbk1-nak-ser172-d52c2-rabbit-monoclonal-antibody/5483?srsltid=AfmBOorjxcTR1k8hZ1M2DfzXmg6RNn3hFiU6-Gv5pRiNefY3pSLHAwVp]<br>NF-κB (Thermo Fischer, 51-0500) : Confirmed species reactivity- Human, Mouse, Non-human primate [https://www.thermofisher.com/antibody/product/NFkB-p65-Antibody-Polyclonal/51-0500]<br>phospho-Ser536-NF-κB (Invitrogen, Ma5-15160, T.849.2) : Confirmed species reactivity- Hamster, Human, Mouse, Non-human primate, Pig, Rat [https://www.thermofisher.com/antibody/product/Phospho-NFkB-p65-Ser536-Antibody-clone-T-849-2-Monoclonal/MA5-15160]<br>IRF3 (Cell Signaling Technology, D6I4C) : Confirmed species reactivity- Human, Monkey [https://www.cellsignal.com/products/primary-antibodies/irf-3-d6i4c-rabbit-monoclonal-antibody/11904?srsltid=AfmBOoplOKeRkN80soZyTbzQiil2lr4QoCv3Agadj32kOtoTgvlg-ggv]<br>phospho-Ser386-IRF3 (Cell Signaling Technology, E7J8G) : Confirmed species reactivity- Human [https://www.cellsignal.com/products/primary-antibodies/phospho-irf-3-ser386-e7j8g-rabbit-monoclonal-antibody/37829?srsltid=AfmBOorhdk01K2BtwsEGcwPgAbFXF-VolJD9BVciRkFW7GKutwA5utPY]<br>activated BAX-6A7 (Santa Cruz, sc23959) : Confirmed species reactivity- mouse, rat and human [https://www.scbt.com/p/bax-antibody-6a7?srsltid=AfmBOop4RGeIwCq9kDX1qykwamNRXpYO-5jGJTNNZl7ttQnhvYMyIDpT]<br>LC3B (Cell Signaling Technology, D11) : Confirmed species reactivity- Human [https://www.cellsignal.com/products/primary-antibodies/lc3b-d11-rabbit-monoclonal-antibody/3868?srsltid=AfmBOopouhVypKqzUG2gl85kycZErhYqo6NfoPQ-mD_y-ukg_om6HnoV]<br>LAMP1 (DSHB, H4A3) : Confirmed species reactivity- Hamster, Human, Primate, Rat [https://dshb.biology.uiowa.edu/H4A3]<br>Cofilin (Invitrogen, PA5-17372) : Confirmed species reactivity- Bovine, Human, Mouse, Non-human primate, Rat [https://www.thermofisher.com/antibody/product/Cofilin-Antibody-Polyclonal/PA5-17372]<br>phospho-Ser3-Cofilin (Invitrogen, PA5-17752) : Confirmed species reactivity- Bovine, Hamster, Human, Mouse, Non-human primate, Pig, Rat [https://www.thermofisher.com/antibody/product/Phospho-Cofilin-Ser3-Antibody-Polyclonal/PA5-17752]<br>GAPDH (Sigma, G9545) : Confirmed species reactivity- mouse, rat, human [https://www.sigmaaldrich.com/US/en/product/sigma/g9545?srsltid=AfmBOoqZNyq1fDNRX4Q3VEFrt3JrclpR72Skz0QVP5JQqYO7Hy3CN1B6]

Eukaryotic cell lines

Policy information about cell lines and Sex and Gender in Research

Cell line source(s)	All cell lines were sourced from ATCC. Human iPSC lines used in our studies were generated following procedures approved by the Commission on Guarantees concerning the Donation and Use of Human Tissues and Cells of the Carlos III Health Institute, Madrid, Spain.
Authentication	All the cells lines used in this study were authenticated by the supplier. iPSC-derived neurons and microglia were validated by immunofluorescence experiments in a previous study.
Mycoplasma contamination	Cells were not tested for mycoplasma contamination regularly. However, periodic testings of cell lines used in the lab, including those in this study, were conducted.
Commonly misidentified lines (See ICLAC register)	No misidentified cell lines were used in the study.

Plants

Seed stocks	N/A
Novel plant genotypes	N/A
Authentication	N/A

Flow Cytometry

Plots

Confirm that:

- [x] The axis labels state the marker and fluorochrome used (e.g. CD4-FITC).
- [x] The axis scales are clearly visible. Include numbers along axes only for bottom left plot of group (a 'group' is an analysis of identical markers).
- [x] All plots are contour plots with outliers or pseudocolor plots.
- [x] A numerical value for number of cells or percentage (with statistics) is provided.

Methodology

Sample preparation	Cells were initially grown in either monocultures or cocultures depending on the experimental paradigm (supplementary Fig. 12d). For collection, cells were washed with PBS, trypsinized, and re-suspended in complete media before being subjected to flow cytometry-assisted sorting.
Instrument	BD FACSymphony S6 sorter
Software	BD FACSDiva. FlowJo v10.1.1
Cell population abundance	RFP+ neuronal cells and RFP- microglia growing in co-cultures were sorted. Cell population percentage post-sorting are mentioned within the plots in Fig. S12d
Gating strategy	Gating strategy was based on cellular expression of RFP.

- [x] Tick this box to confirm that a figure exemplifying the gating strategy is provided in the Supplementary Information.
