# Supplementary material for: α-Synuclein aggregates induce mitochondrial damage and trigger innate immunity to drive neuron–microglia communication
Source: Nat Commun. 2026 May 15;17:6462. doi: 10.1038/s41467-026-73136-7 (PMC13376753; doi:10.1038/s41467-026-73136-7)
Supplement: Supplementary file 3 — Transparent Peer Review file [file 41467_2026_73136_MOESM3_ESM.pdf]

nature portfolio

Peer Review File

α-Synuclein aggregates induce mitochondrial damage and trigger innate immunity to drive neuron–microglia communication

Corresponding Author: Professor Chiara Zurzolo

This file contains all reviewer reports in order by version, followed by all author rebuttals in order by version.

Version 0:

Reviewer comments:

Reviewer #1

(Remarks to the Author)
The manuscript of Chakraborty et al. addresses an important question regarding the mechanisms supporting the spread of neurodegenerative diseases through the brain. In their previous work, the team has already established TNT connections as a means of disease spreading. The present study takes a significant step forward by identifying a mechanistic link between (α-Syn) aggregation and TNT formation.

The study demonstrates that formation of (α-Syn) aggregates leads to mitochondrial damage which subsequently triggers cGAS–STING–NF-κB–IRF3 activation and promotes TNT formation between neurons and microglia.

Notably, since TNTs have previously been shown to support transfer of (α-Syn) aggregates and of damaged mitochondria from neurons to microglia, these findings offer compelling mechanistic evidence for disease propagation.

As STING activation has been reported in several neurodegenerative diseases including Alzheimer, Parkinson, Huntington, and amyotrophic lateral sclerosis, the findings of this study, concerning Synucleinopathies like Parkinson and dementia with Lewy bodies, are likely to apply to the other neurodegenerative diseases as well.

Comments

1. On the basis of cGAS–STING–NF-κB–IRF3 activation as a marker of the innate immune response, the authors present their findings as the first mechanistic evidence that neurodegeneration-associated inflammation actively drives TNT-mediated neuron–microglia communication. The is obviously the strong point and main contribution of the manuscript.

However, given that STING-mediated inflammation is central to the manuscript’s conclusions, it would warrant additional experimental data using complementary experimental approaches to strengthen and validate the findings.

2. The authors report that the addition of α-Syn aggregates to cells results in their localization to mitochondria. While this observation is supported by previous publications, the cell imaging data shown do not appear particularly convincing. To strengthen this point, It would be appropriate to include controls using unrelated proteins to determine whether their localization differs from that of α-Syn aggregates.
Furthermore, it is unclear whether the authors intend to stress out the novelty of the experimental approach using exogenous pre-formed fibrils of α-Syn. If this is a key point, it should be clearly stated. Otherwise, because this finding is neither novel nor central to the manuscript main conclusions, it is recommended that the authors either provide appropriate controls or consider moving these data to the supplementary section.

3. The authors compare the effects of α-Syn in neurons and in microglial cells. However, an appropriate comparison would rely on similar amounts of transferred α-Syn. Was this quantified and how?

4. The experiments were conducted using both primary neurons and microglial cells and iPSCs-derived cells. The authors should clarify the specific contribution of each experimental model - whether they serve merely to replicate findings - or

provide distinct, biologically relevant insights. At present, this distinction is not clearly addressed in either in the results or discussion sections.

5. Since the authors suggest that their findings highlight new potential therapeutic targets, it would be valuable to know whether any of these targets were experimentally tested and, if so, what the outcomes were.

Overall, this is an interesting and well-written study highlighting the role of cGAS–STING–NF-κB–IRF3 pathway activation in mediating (α-Syn)-dependent TNT formation. The research team's strong expertise in cell imaging is clearly an asset and contributes significantly to the quality of the data presented. However, since the conclusions rely primarily on imaging techniques, it would be beneficial for the authors to include complementary experimental approaches to further substantiate their findings.

Reviewer #2

(Remarks to the Author)
Chakraborty and colleagues present a study investigating effects of alpha-Synuclein (a-Syn) on mitochondrial function, inflammation. Their data shows association of aggregated a-Syn on mitochondria, mitochondrial dysfunction leading to mtDNA release and inflammation – this the authors suggest is via MOMP. The inflammatory effect leads to TNT formation and mitochondrial transfer to microglia, whereupon the authors propose that mitochondria are degraded, and mitochondrial transfer promotes inflammation in neighbouring microglia. The authors present an interesting study, with clear potential pathological relevance, the data largely support their conclusions. Nonetheless, some aspects could do with orthogonal validation and additional controls to support the authors model. These are detailed below:

1 – As far as I can determine, the authors perform all their expts. with aggregated Syn. I think it important to assess whether the effects of Syn on neuronal cells is dependent on its aggregation or not (to implicate a disease relevance of these effects). While I am not suggesting the whole study needs this, at least looking in neuronal cells to assess whether addition of non-aggregated Syn has any impact on mitochondrial function, inflammation would go a long way to supporting the authors' model that the effects determined are specific to aggregated Syn.

2 – The authors propose that canonical MOMP (mediated via BAX/BAK) is required for the inflammatory effects they observe (and subsequent impact on TNT formation), this is never actually tested but should be – specifically asking do SH-SY5Y and HMC3 cells deficient in the mitochondrial pathway (can be generated through BAX/BAK deletion of anti-apoptotic BCL-XL overexpression) display inhibited inflammation (STING, NFkB activation), reduced TNT formation ? (supporting their model).

3 – The mechanism of bystander inflammation is unclear, is this dependent on mitochondria transferred, effects on recipient cell mitochondria and, or other aspects for instance cGAMP transfer. While I realise this is not necessarily trivial to decipher, some attempt to address this key question could be made by manipulating signaling mechanisms in donors (e.g. blocking cGAS activity or MOMP) vs acceptors, then monitoring inflammatory output in the receivers, should allow general principles to be established.

Reviewer #3

(Remarks to the Author)
The manuscript by Chakraborty et al. presents an elegant and comprehensive study investigating how α-synuclein (α-Syn) aggregates impair mitochondrial function, induce mtDNA release, and activate the cGAS–STING–NF-κB innate immune pathway to promote tunnelling nanotube (TNT)-mediated neuron–microglia communication. The authors provide compelling evidence using both cell lines and iPSC-derived neurons and microglia, thereby strengthening the translational relevance of their findings. The discovery that damaged mitochondria are transferred from neurons to microglia for degradation adds a novel dimension to the role of microglia in neuroprotection. The work identifies mitochondrial damage and innate immune activation as central drivers of TNT biogenesis and α-Syn propagation. However, several aspects of the work could be improved as follows:

Extent of microglial neuroprotection: While the data indicate that microglia degrade damaged mitochondria, it remains unclear whether this process has a net beneficial effect on neuronal survival. Quantitative assays of neuronal viability or function after co-culture with microglia would be necessary to clarify the protective relevance of this pathway.

Specificity of STING activation: The data convincingly show that α-Syn induces STING pathway activation. However, the possibility of additional DNA or RNA sensors contributing to the observed effects should be discussed. For example, is there any contribution of TLR9 or RIG-I–like receptors to the response?

In addition, it is possible that the STING inhibitor H151 prevents TNT formation independently of mtDNA content/release as this could affect basal IFN signalling and potentially TNTs. Experiments aimed at depleting cells from mtDNA (through ddC or EtBr) should be included to enforce the role of these nucleic acids in the observed phenotypes.

Role of secreted factors: The manuscript demonstrates a bystander activation of microglia, but it is not clear whether this is mediated primarily by TNT-dependent transfer, mtDNA release, or soluble cytokines. Some clarification through additional experiments would help disentangle these mechanisms.

Extended figures 3: The increased Nf-kb nuclear localization is not clear from this figure.
The suggested accumulation of DNA is only seen by IF. Also, there is no clear demonstration that it is of mitochondrial in origin. Mitochondria-specific q PCR after fractionation and appropriate DNase/RNase controls should be added to all IF experiments.

In Figure 8, gene expression analysis and biochemical confirmation of type I IFN and inflammatory pathway activation would better corroborate proposed effects of transferred damaged mitochondria on the receiving cells.

Overall, positive, and negative controls are lacking across experiments and should be included, for example for STING activation and IRF3/NFkB translocation.

STING-dependent TNT formation and mitochondrial transfer in models of Parkinson's disease. A discussion of how these pathways may operate in the complex brain microenvironment should be expanded.

Reviewer #4

(Remarks to the Author)
I co-reviewed this manuscript with one of the reviewers who provided the listed reports. This is part of the Nature Communications initiative to facilitate training in peer review and to provide appropriate recognition for Early Career Researchers who co-review manuscripts.

Reviewer #5

(Remarks to the Author)
I co-reviewed this manuscript with one of the reviewers who provided the listed reports. This is part of the Nature Communications initiative to facilitate training in peer review and to provide appropriate recognition for Early Career Researchers who co-review manuscripts.

Version 1:

Reviewer comments:

Reviewer #1

(Remarks to the Author)
The authors appropriately addressed the questions that were raised and made the corresponding few adjustments that were requested. Additionally, the inclusion of a detailed working model based on the obtained data is appreciated, as it helps to clearly highlight the main results and implications of this very interesting study.

Reviewer #2

(Remarks to the Author)
The authors have comprehensively addressed all my comments

Reviewer #3

(Remarks to the Author)
The inclusion of appropriate controls, inhibitor studies for alternative sensors and the assessment of bystander inflammation has significantly clarified the pathway. However, for certain claims biochemical proof is still missing.

Microglial Neuroprotection: The addition of K63-ubiquitin chains/TOMM20 staining in Figure 7j provides a mechanistic link to the protective role of microglia. While a survival assay (like LDH release or similar) would have provided a direct proof of neuroprotection, the significant reduction in ubiquitinated mitochondrial clusters in neurons indicates improved neuronal homeostasis.

The authors have addressed the concerns regarding alternative nucleic acid sensors. The new data using TLR9 and RIG-1 inhibitors (Fig. S9), alongside the neuronal-specific cGAS inhibition (Fig. S12), narrows the inflammatory signaling pathway to the cGAS-STING axis.

Validation of Mitochondrial DNA in the Cytosol: While the authors provide imaging data and a BAX inhibition assay to support the mitochondrial origin of cytosolic DNA, this remains an inference. DAPI has a lower affinity for small, extranuclear DNA fragments and is not a definitive marker for nuclear or mitochondrial origin. A cytosolic qPCR (measuring mitochondrial genes vs nuclear markers in the isolated cytosolic fraction) would be needed to confirm mtDNA release. The argument against performing mtDNA depletion based on safety regulations for EtBr and ddC is unconvincing. While EtBr does require specific handling, 2',3'-dideoxycytidine (ddC) is a reagent and nucleoside analogue commonly used in the field for generating mtDNA-depleted cells without such regulatory barriers. Given that ddC-mediated depletion is the standard for

proving the mtDNA-dependency of the STING pathway, the authors should utilize this more accessible alternative for a stronger functional evidence.

Open Access This Peer Review File is licensed under a Creative Commons Attribution 4.0 International License, which permits use, sharing, adaptation, distribution and reproduction in any medium or format, as long as you give appropriate credit to the original author(s) and the source, provide a link to the Creative Commons license, and indicate if changes were made.
In cases where reviewers are anonymous, credit should be given to 'Anonymous Referee' and the source.
The images or other third party material in this Peer Review File are included in the article's Creative Commons license, unless indicated otherwise in a credit line to the material. If material is not included in the article's Creative Commons license and your intended use is not permitted by statutory regulation or exceeds the permitted use, you will need to obtain permission directly from the copyright holder.
To view a copy of this license, visit https://creativecommons.org/licenses/by/4.0/

We would like to thank all the reviewers for their valuable comments and inputs towards our study that pushed us to address as many concerns and questions as were in our capacities. We believe that our revised manuscript has thus been strengthened. We address the individual points below.

Reviewer #1 (Remarks to the Author):

The manuscript of Chakraborty et al. addresses an important question regarding the mechanisms supporting the spread of neurodegenerative diseases through the brain. In their previous work, the team has already established TNT connections as a means of disease spreading. The present study takes a significant step forward by identifying a mechanistic link between (α-Syn) aggregation and TNT formation.

The study demonstrates that formation of (α-Syn) aggregates leads to mitochondrial damage which subsequently triggers cGAS–STING–NF-κB–IRF3 activation and promotes TNT formation between neurons and microglia.

Notably, since TNTs have previously been shown to support transfer of (α-Syn) aggregates and of damaged mitochondria from neurons to microglia, these findings offer compelling mechanistic evidence for disease propagation.

As STING activation has been reported in several neurodegenerative diseases including Alzheimer, Parkinson, Huntington, and amyotrophic lateral sclerosis, the findings of this study, concerning Synucleinopathies like Parkinson and dementia with Lewy bodies, are likely to apply to the other neurodegenerative diseases as well.

Comments

1. On the basis of cGAS–STING–NF-κB–IRF3 activation as a marker of the innate immune response, the authors present their findings as the first mechanistic evidence that neurodegeneration-associated inflammation actively drives TNT-mediated neuron–microglia communication. The is obviously the strong point and main contribution of the manuscript.

However, given that STING-mediated inflammation is central to the manuscript's conclusions, it would warrant additional experimental data using complementary experimental approaches to strengthen and validate the findings.

We thank the reviewer for this comment. While we agree that orthogonal validation of activation of the STING cascade would strengthen our findings, we could not manage to get the commercially available antibodies (used here in this study for immunofluorescence) work for western blots. However, we had performed RT-PCR to measure the levels of transcripts that would canonically be upregulated via the cGAS-STING pathway (IRF3-dependent type I interferon response). We observe a robust increase in the expression levels of both IFN-α and IFN-β (**updated Fig S5q, r**), underscoring an activation of the pathway. We believe this result provides a strong support towards the activation of cGAS-STING pathway in the presence of α-Syn aggregates.

2. The authors report that the addition of $\boldsymbol{\alpha}$-Syn aggregates to cells results in their localization to mitochondria. While this observation is supported by previous publications, the cell imaging data shown do not appear particularly convincing. To strengthen this point, It would be appropriate to include controls using unrelated proteins to determine whether their localization differs from that of $\boldsymbol{\alpha}$-Syn aggregates.
Furthermore, it is unclear whether the authors intend to stress out the novelty of the experimental approach using exogenous pre-formed fibrils of α-Syn. If this is a key point, it should be clearly stated. Otherwise, because this finding is neither novel nor central to the manuscript main conclusions, it is recommended that the authors either provide appropriate controls or consider moving these data to the supplementary section.

We thank the reviewer for a critical assessment of this aspect. Several studies had previously reported that α-Synuclein aggregates localize to mitochondria in both murine cells, as well as human iPSC-derived neurons. The association of aggregated species of α-Synuclein with mitochondria resulted in compromised organellar functions. As such, our observations in human neuronal and microglial cell lines are not stand-alone novel (*Nakamura et al., Journal of Neuroscience, 2008; Martinez et al., Archives of Biochemistry and Biophysics, 2018; Di Maio et al., Science Translational Medicine, 2016; Choi et al., Nature Neuroscience, 2022*). These experiments were conducted to validate whether the exogenous PFFs of α-Synuclein we used also recapitulated previous findings. We agree with the reviewer that these results are not central to the manuscript's conclusions but rather provide a foundation upon which the rest of the work was built upon. Keeping this into consideration, we have moved these results to the supplementary information section and updated the main manuscript text in lines 132-152 (updated Fig. S1a-d).

3. The authors compare the effects of $\boldsymbol{\alpha}$-Syn in neurons and in microglial cells. However, an appropriate comparison would rely on similar amounts of transferred $\boldsymbol{\alpha}$-Syn. Was this quantified and how?

We thank the reviewer for this comment. Indeed, to be able to compare the effect of α-Synuclein aggregates on organellar health in neuronal and microglial cells, the overall load of α-Synuclein should be similar between the two cell types. However, we have previously observed that the overall load of α-Synuclein aggregates is relatively higher in neuronal cells than in microglia (number of α-Syn particles normalized to cell area) [*Chakraborty et al., Nature communications, 2026, In Press; Figure R1*]. From our previous studies, such an observation can be attributed to the impaired ability of neuronal cells to clear α-Syn aggregates compared to microglia, thereby resulting in a higher burden. Taking this into consideration, the main aims of our study were to elucidate the detrimental effects of these aggregates on mitochondrial health and consequential activation of the innate immune pathway cGAS-STING. As such, our goal was to compare cells that were treated with aggregates or not, and not necessarily a direct comparison of neuronal cells and microglia. We have addressed this point in the main manuscript file by rephrasing our results section (lines 173-175) to avoid any confusion for the readers.

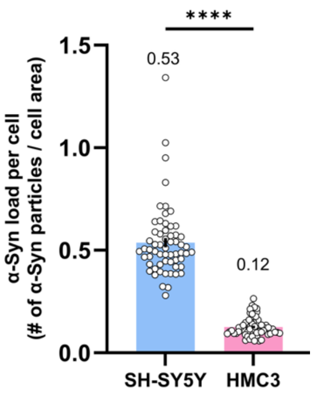

***Figure R1**: Quantification of α-Syn burden in neuronal and microglial cells after 16h of aggregate exposure highlights a relatively higher aggregate load (number of α-Syn particles per unit area of the cell) in neuronal cells than microglia.*

***(Note to the editor**: This data is from Supplementary Figure S5b of our manuscript entitled "Impaired α -Synuclein aggregate clearance in neuronal cells drive their spread to microglia through tunneling nanotubes"; Article ID: 41467_2026_69930_Article; Manuscript ID: NCOMMS-25-27356, which is currently in press at Nature Communications).*

4. The experiments were conducted using both primary neurons and microglial cells and iPSCs-derived cells. The authors should clarify the specific contribution of each experimental model - whether they serve merely to replicate findings - or provide distinct, biologically relevant insights. At present, this distinction is not clearly addressed in either in the results or discussion sections.

We thank the reviewer for pointing out this critical aspect. The use of iPSC-derived neurons and microglia were to reproduce our findings from cell lines in a physiologically relevant model system. We observe similar response of these cells to α-Syn aggregates added exogenously, which not only recapitulates our findings from cell lines, but also solidifies the biology of α-Syn aggregates-mitochondria interplay as had been reported by other groups, notably that of Dr. Sonia Gandhi from the Francis Crick Institute (*Choi et al., Nature Neuroscience, 2022*). We have now clarified this point in the manuscript file in both the results (lines 137-139), and the discussion sections (lines 474-476).

5. Since the authors suggest that their findings highlight new potential therapeutic targets, it would be valuable to know whether any of these targets were experimentally tested and, if so, what the outcomes were.

We thank the reviewer for raising this point. Since the discovery of cGAS-STING pathway in responding to intracellular nucleic acids, several studies have implicated the involvement of this cascade in healthy aging and cellular senescence, as well as several neurodegenerative diseases. In vivo studies have shown that pharmacological inhibition of STING, or its genetic deletion is neuroprotective in an othwerwise inflammatory environment. These findings highlight a potential route for therapeutic interventions. However, the aim of our study was to uncover a fundamental principle governing intercellular communication in a minimal in vitro environment. Extrapolating these results would warrant the validation of the cGAS-STING pathway in aggregate spread in vivo. We hope that future studies along this direction will yield significant new knowledge, which can then be leveraged for therapeutic purposes. We have rephrased our manuscript accordingly to highlight this aspect (lines 548-553).

Overall, this is an interesting and well-written study highlighting the role of cGAS–STING–NF-κB–IRF3 pathway activation in mediating (α-Syn)-dependent TNT formation. The research team's strong expertise in cell imaging is clearly an asset and contributes significantly to the quality of the data presented. However, since the conclusions rely primarily on imaging techniques, it would be beneficial for the authors to include complementary experimental approaches to further substantiate their findings.

Reviewer #2 (Remarks to the Author):

Chakraborty and colleagues present a study investigating effects of alpha-Synuclein (a-Syn) on mitochondrial function, inflammation. Their data shows association of aggregated a-Syn on mitochondria, mitochondrial dysfunction leading to mtDNA release and inflammation – this the authors suggest is via MOMP. The inflammatory effect leads to TNT formation and mitochondrial transfer to microglia, whereupon the authors propose that mitochondria are degraded, and mitochondrial transfer promotes inflammation in neighbouring microglia. The authors present an interesting study, with clear potential pathological relevance, the data largely support their conclusions. Nonetheless, some aspects could do with orthogonal validation and additional controls to support the authors model. These are detailed below:

1 – As far as I can determine, the authors perform all their expts. with aggregated Syn. I think it important to assess whether the effects of Syn on neuronal cells is dependent on its aggregation or not (to implicate a disease relevance of these effects). While I am not suggesting the whole study needs this, at least looking in neuronal cells to assess whether addition of non-aggregated Syn has any impact on mitochondrial function, inflammation would go a long way to supporting the authors' model that the effects determined are specific to aggregated Syn.

We thank the reviewer for their comment. Indeed, previous studies had reported the detrimental effects of aggregated α-Syn on mitochondria in both murine and human cells. To address this aspect, we performed new experiments with monomeric forms of α-Syn. Upon exposure of both neuronal and microglial cells to these monomeric species, we observe no change in the overall morphology of mitochondria (updated Fig. S2a-c), as opposed to severe fragmentation observed in the presence of aggregated α-Syn. We next assessed the membrane potential of these organelles and observed an increase in the mean fluorescence intensity of TMRM in both the cell types (as opposed to drastic reductions observed for aggregated α-Syn) – although the difference was not statistically significant in neuronal cells (updated Fig. S2d-f). An increase in mitochondrial membrane potential has been previously observed in cells exposed to mild stressors, highlighting that monomeric α-Syn does not damage mitochondrial state. In line with these observations, we also do not observe activation of STING or translocation of NF-κB to the nucleus (updated Fig. S4). Taken together with our previous observations, these new results highlight that α-Syn aggregates, and not monomers, can damage mitochondria to induce an innate immune response. These new findings have been incorporated in

updated Fig. S2 and S4. Main manuscript text has been revised in lines 175-180, 234-236, and 248-250.

2 – The authors propose that canonical MOMP (mediated via BAX/BAK) is required for the inflammatory effects they observe (and subsequent impact on TNT formation), this is never actually tested but should be – specifically asking do SH-SY5Y and HMC3 cells deficient in the mitochondrial pathway (can be generated through BAX/BAK deletion of anti-apoptotic BCL-XL overexpression) display inhibited inflammation (STING, NFkB activation), reduced TNT formation ? (supporting their model).

We thank the reviewer for raising this important point that pertains to the central conclusion of our study. We have added new results that demonstrate the involvement of BAX-mediated mtDNA release in inflammation and formation of intercellular connections. Using a pharmacological inhibitor of BAX oligomerization on mitochondrial surfaces BAI-1, we demonstrate that mtDNA is not released in the cytoplasm [updated Fig. 3h-l, main manuscript text lines 224-228]. In addition, inhibiting BAX oligomerization on mitochondrial surfaces resulted in shunted activation of STING (updated Fig. S6a-c), translocation of NF-κB to the nucleus (updated Fig. S6d-f), and no upregulation of intercellular connections even in the presence of α-Syn aggregates (updated Fig. S6g-i). These findings further support our model of BAX-dependent mtDNA release as a contributing factor of inflammation and intercellular connections. Main manuscript text has been revised in lines 262-267.

3 – The mechanism of bystander inflammation is unclear, is this dependent on mitochondria transferred, effects on recipient cell mitochondria and, or other aspects for instance cGAMP transfer. While I realise this is not necessarily trivial to decipher, some attempt to address this key question could be made by manipulating signaling mechanisms in donors (e.g. blocking cGAS activity or MOMP) vs acceptors, then monitoring inflammatory output in the receivers, should allow general principles to be established.

We thank the reviewer for raising this important point. We appreciate the novelty of non-cell autonomous regulation of cellular functions in the field of cell biology of intercellular communications. Several studies in the past have reported such dynamics of intercellular communication. To address the players regulating bystander inflammation, we performed new co-culture experiments of naïve neuronal cells with microglia, and α-Syn-exposed neuronal cells treated or not with cGAS inhibitor (G140), STING inhibitor (H-151) and NF-κB inhibitor (JSH-23) with microglia. We observed a significant rescue of the bystander inflammatory phenotypes (NF-κB and IRF3 translocation to the nucleus) in microglia upon inhibition of cGAS in neuronal cells (updated Fig. S12a, b; g, j; m, p), and not upon STING or NF-κB inhibitions (updated Fig. S12 c, d; h, k; n, q pertains to STING inhibition, and updated Fig. S12e, f; i, l; o, r pertains to NF-κB inhibition). These results highlight a possible role of cGAMP transfer from neuronal cells to microglia as a major determinant of microglial inflammation. Main manuscript text has been revised in lines 439-450.

Reviewer #3 (Remarks to the Author):

The manuscript by Chakraborty et al. presents an elegant and comprehensive study investigating how α-synuclein (α-Syn) aggregates impair mitochondrial function, induce mtDNA release, and activate the

cGAS–STING–NF-κB innate immune pathway to promote tunnelling nanotube (TNT)-mediated neuron–microglia communication. The authors provide compelling evidence using both cell lines and iPSC-derived neurons and microglia, thereby strengthening the translational relevance of their findings. The discovery that damaged mitochondria are transferred from neurons to microglia for degradation adds a novel dimension to the role of microglia in neuroprotection. The work identifies mitochondrial damage and innate immune activation as central drivers of TNT biogenesis and α-Syn propagation. However, several aspects of the work could be improved as follows:

1. Extent of microglial neuroprotection: While the data indicate that microglia degrade damaged mitochondria, it remains unclear whether this process has a net beneficial effect on neuronal survival. Quantitative assays of neuronal viability or function after co-culture with microglia would be necessary to clarify the protective relevance of this pathway.

We thank the reviewer for raising this important point. Our studies in the past, as well as those from the lab of Prof. Michael Heneka (*Chakraborty et al., Cell Death and Disease, 2023; Scheiblich et al., Neuron, 2024*) have illustrated the roles of TNTs between neurons and microglia in being neuroprotective in conditions stressful for neurons. To address this comment, we had a couple of initial thoughts – (a) to perform electrophysiological recordings of neurons in the presence of microglia, and (b) to assess the functional state of mitochondria using TMRM. However, both such assays have previously demonstrated that microglia are indeed neuroprotective (Scheiblich et al., Neuron, 2024). The central conclusion of that study is that neuronal functions are rescued owing to the transfer of functional mitochondria to neurons. However, we tried to address whether transfer of damaged mitochondria from α-Syn-exposed neuronal cells to microglia is also protective (in essence, the other side of the same coin). Since we show that the overall flux of mitophagy is compromised in neuronal cells, we hypothesized that co-culture with microglial cells will reduce the burden of damaged mitochondria in neuronal cells, if indeed dysfunctional mitochondria are sent out to microglia. Towards this, we performed immunostaining against K-63 ubiquitin chains and assessed the extent of its overlap with TOMM20. We find a significant reduction in the level of K63-ubiquitinated mitochondria in α-Syn exposed neuronal cells when in co-culture with mitochondria. This suggests a protective aspect of mitochondrial transfer from neuronal cells to microglia. These findings have been added in updated Fig. 7j. Main manuscript text has been revised in lines 407-412.

2. Specificity of STING activation: The data convincingly show that α-Syn induces STING pathway activation. However, the possibility of additional DNA or RNA sensors contributing to the observed effects should be discussed. For example, is there any contribution of TLR9 or RIG-I–like receptors to the response?

We thank the reviewer for raising this prospect. It is indeed plausible that additional nucleic acid sensors could contribute to an inflammatory response. Several studies in the recent past have implicated the release of mitochondrial RNA in mounting an innate immune response (*Dhir et al., Nature, 2018; Gokhale et al., Science, 2024*). Additionally, mtDNA can be targeted for endo-lysosomal disposal which could then be recognized by TLR9. To address this, we have performed new experiments using TLR9 inhibitor (TLR9-IN-1) and RIG-1 inhibitor (RIG012) assessing the state of inflammation and intercellular

connections. We observe no significant involvement of TLR9 in α-Syn exposed cells in mediating the translocation of NF-κB to the nucleus, or in the formation of intercellular connections (updated Fig. S9). Upon inhibition of RIG-1, we only observe a very slight reduction in intercellular connections and nuclear NF-κB occupancy, highlighting that neither of these nucleic acid sensors are major players in α-Syn-induced innate immune activation. However, other potential nucleic acid sensors like AIM2 or ZBP1 could still have a role to play in the formation of intercellular connections, thereby warranting further studies. Main manuscript text has been revised in lines 321-333.

3. In addition, it is possible that the STING inhibitor H151 prevents TNT formation independently of mtDNA content/release as this could affect basal IFN signalling and potentially TNTs. Experiments aimed at depleting cells from mtDNA (through ddC or EtBr) should be included to enforce the role of these nucleic acids in the observed phenotypes.

We thank the reviewer for raising this concern. H-151 treatment can affect basal type-I IFN signaling. However, our previous results (Fig 5a-c) show that H151 treatment alone does not reduce connections below control conditions. Together with our gene expression results, we observe that both neuronal and microglial cells were relatively more robust in upregulating the expression of cytokines than interferons in response to α-Syn aggregates. We would have liked to also assess the role of IRF3 in the formation of intercellular connections, but the unavailability of a specific inhibitor of IRF3, and technical difficulties in transfecting HMC3 microglia prevented us from doing so.

Regarding depleting cells of mtDNA, we appreciate the relevance of this approach. However, safety regulations regarding the use of certain chemicals (such as EtBr) prevented us from doing so. Nevertheless, the lack of DAPI positivity of cytosolic dsDNA particles allow us to infer a lack of micronuclei in the cytoplasm, and that the origin of dsDNA particles is mitochondrial. In addition, dsDNA signals are mostly contained within mitochondria when cells were treated with a pharmacological inhibitor of BAX (updated Fig. 3h, i), thereby allowing us to ascertain mitochondrial origin of cytosolic dsDNA. A sentence has been added (lines 216-218) in the revised manuscript addressing this point.

4. Role of secreted factors: The manuscript demonstrates a bystander activation of microglia, but it is not clear whether this is mediated primarily by TNT-dependent transfer, mtDNA release, or soluble cytokines. Some clarification through additional experiments would help disentangle these mechanisms.

We thank the reviewer for raising this important concern. As mentioned in the response to the third comment of reviewer #2, we believe the importance of addressing the potential players regulating bystander inflammation. We have now added new experiments in the study (updated Fig. S12) to address the route of bystander inflammation in microglial cells. We observe that inhibition of neuronal cGAS (but not inhibitions of STING or NF-κB) is sufficient to prevent the mounting of a robust bystander inflammatory response, highlighting a plausible role of cGAMP transfer from α-Syn-burdened neuronal cells to microglia in the process. Inhibition of molecular players downstream of cGAS (such as STING or NF-κB) failed to prevent bystander inflammation possibly because of the second messenger cGAMP still being produced in neuronal cells, capable of being transferred. Although our results provide preliminiary

evidence towards this process, it certainly provides a foundation for future experiments with genetic deletion approaches, and use of physiologically advanced systems. We highlight this, and the limitation of our findings in the discussion of the manuscript.

5. Extended figures 3: The increased Nf-kb nuclear localization is not clear from this figure.
The suggested accumulation of DNA is only seen by IF. Also, there is no clear demonstration that it is of mitochondrial in origin. Mitochondria-specific q PCR after fractionation and appropriate DNase/RNase controls should be added to all IF experiments.

We thank the reviewer for this comment. We have now updated the figure pertaining to NF-κB nuclear translocation. Regarding the origin of dsDNA particles in the cytosol, we agree with the reviewer that this can be achieved in two different ways under non-infectious conditions – (a) release of nuclear DNA in the form of micronuclei, or (b) release of mtDNA from the mitochondria. In both cases, nucleic acid sensors can recognize dsDNA and mount an innate immune response. To the best of our knowledge, most of micronuclei in the cytoplasm would also be positive for DAPI, owing to a strong affinity of the dye towards nuclear DNA sequences. However, we observe dsDNA particles in the cytosol that are consistently negative for DAPI. In addition, upon prevention of BAX oligomerization on mitochondrial membranes, we observe a robust prevention of dsDNA+ particles released in the cytoplasm (updated Fig. 3h, i). Together, these observations allowed us to infer a mitochondrial origin of cytosolic dsDNA particles. A sentence has been added (lines 216-218) in the revised manuscript addressing this point.

6. In Figure 8, gene expression analysis and biochemical confirmation of type I IFN and inflammatory pathway activation would better corroborate proposed effects of transferred damaged mitochondria on the receiving cells.

We thank the reviewer for this suggestion. We agree that alterations in microglial gene expression corroborates our findings on bystander inflammation. To address this, we co-cultured RFP-expressing naïve or α-Syn-exposed neuronal cells with microglia and eventually sorted non-RFP+ microglia using flow cytometry. Upon analyzing the expression of type I interferons IFNα and IFNβ in these cells, we observed a near 2-fold increase in the expression of these genes in microglia co-cultured with α-Syn-burdened neuronal cells. We expected a low level of gene expression changes in this experimental paradigm relative to microglia directly exposed to α-Syn owing to the differences in magnitude of inflammatory activation via cell autonomous and non-cell autonomous manners. Nevertheless, our findings highlight bystander inflammation to potentially alter transcriptional signatures of microglia. These results have now been added in updated Fig.S11d, e. Main manuscript file has been revised (lines 430-435) addressing this point.

Overall, positive, and negative controls are lacking across experiments and should be included, for example for STING activation and IRF3/NFkB translocation.

We thank the reviewer for this comment. We have now incorporated appropriate controls for the experiments:

Updated Fig. 4a-c; and g-k: diABZI (positive control to assess STING activation) has been added.

Updated Fig. S5a-c: LPS (positive control for NF-κB translocation to the nucleus) has been added.

Updated Fig. S5i-k: diABZI (positive control for STING activation-mediated IRF3 translocation the the nucleus) has been added.

STING-dependent TNT formation and mitochondrial transfer in models of Parkinson's disease. A discussion of how these pathways may operate in the complex brain microenvironment should be expanded.

We thank the reviewer for this comment. We have now updated the manuscript accordingly in the discussion section (lines 510-521).

Reviewer #4 (Remarks to the Author):

I co-reviewed this manuscript with one of the reviewers who provided the listed reports. This is part of the Nature Communications initiative to facilitate training in peer review and to provide appropriate recognition for Early Career Researchers who co-review manuscripts.

We thank the reviewer for being a part of the review process and dedicating time in doing so.

Reviewer #5 (Remarks to the Author):

I co-reviewed this manuscript with one of the reviewers who provided the listed reports. This is part of the Nature Communications initiative to facilitate training in peer review and to provide appropriate recognition for Early Career Researchers who co-review manuscripts.

We thank the reviewer for being a part of the review process and dedicating time in doing so.

Response to Reviewer #3

We thank the reviewer for their thoughtful and constructive comments. We have carefully considered the points raised and provide detailed responses below.

Comment 1:

The reviewer raises the possibility that nuclear DNA (nucDNA) may be released into the cytosol following α-synuclein treatment and could contribute to cGAS–STING activation.

Response:
We agree with the reviewer that nuclear DNA release into the cytosol may occur under certain stress conditions, including α-synuclein exposure. However, our current dataset provides both direct and functional evidence supporting a mitochondrial origin of the immunogenic DNA driving cGAS–STING activation in our system.

Specifically:

- We detect cytosolic DNA using targeted mtDNA labeling approaches (via TFAM).
- These observations are made in the context of α-synuclein-induced mitochondrial damage and BAX-dependent mitochondrial outer membrane permeabilization (MOMP), a well-established mechanism for mtDNA release (McArthur et al., *Science*, 2018; Riley et al., *EMBO J.*, 2018).
- Importantly, inhibition of BAX markedly reduces STING activation and downstream inflammatory responses, consistent with previous reports linking MOMP-driven mtDNA release to cGAS–STING activation (Rongvaux et al., *Cell*, 2014; Graziani et al., Endothelial RRM2B-dependent mitochondrial DNA signalling drives doxorubicin cardiotoxicity, BioRxiv, 2026).

Together, these results demonstrate a clear functional dependency on mitochondrial DNA release.

Importantly, if chromatin-derived DNA were a major contributor to STING activation in this context, inhibition of BAX would not be expected to produce the strong rescue phenotype that we observe. Rather, only a partial effect would be anticipated. The robust suppression of inflammation and intercellular connection formation upon BAX inhibition therefore strongly supports mtDNA as the principal driver.

While we acknowledge that a potential contribution of nuclear DNA is of mechanistic interest, we do not consider that it alters the central conclusions of the study. We will incorporate this point into the Discussion as an important direction for future investigation.

Comment 2:

The reviewer suggests performing cytosolic fractionation followed by qPCR to distinguish between mitochondrial and nuclear DNA.

Response:
We appreciate this suggestion. However, we respectfully consider that cytosolic fractionation followed by qPCR would provide limited additional mechanistic insight in the context of our study.

First, even if nuclear DNA were detected in the cytosol, this would not alter the functional interpretation of our data, which already demonstrate that STING activation depends on mitochondrial DNA release.

Second, Cytosolic fractionation approaches used to quantify mitochondrial DNA are technically challenging and require stringent controls to ensure fraction purity, as they are inherently susceptible to contamination from nuclear and mitochondrial compartments. This limitation can complicate the reliable distinction between nuclear and mitochondrial DNA species and may introduce ambiguity in data interpretation.

For these reasons, we believe that such experiments would not substantially strengthen the central conclusions of the manuscript.

Comment 3:

The reviewer suggests depletion of mitochondrial DNA (e.g., using ddC) to assess its role in STING activation.

Response:
We agree that mtDNA depletion is, in principle, an approach to assess the contribution of mitochondrial DNA. However, this strategy is not without important confounding factors.

While ddC is often considered a safer alternative to ethidium bromide (EtBr), recent evidence indicates that ddC treatment itself can induce ferroptosis via activation of the cGAS–STING pathway (Juan Hui et al., *Journal of Translational Medicine*, 2026). Therefore, ddC may directly influence the pathway under investigation, complicating interpretation of results.

Thus, although feasible, mtDNA depletion using ddC introduces a significant confounding variable when attempting to dissect the relative contributions of mitochondrial versus nuclear DNA in the formation of intercellular connections, provided our results demonstrating the cGAS-STING pathway to positively regulate the formation of functional TNTs.

Comment 4:

The reviewer raises the broader possibility that chromatin-derived DNA could contribute to innate immune activation in this context.

Response:
We agree that cytosolic chromatin fragments, particularly in the form of micronuclei (MN), have been implicated in cGAS–STING activation in contexts of genotoxic stress, genomic instability, and cellular senescence (Dou et al., *Nature*, 2017; Glück et al., *Nat Cell Biol*, 2017; Mackenzie et al., *Nature*, 2017).

However, several key distinctions suggest that this mechanism is unlikely to be a major contributor in our system:

1. Context and kinetics

MN formation is typically associated with chronic genotoxic stress and delayed responses. For example, STING activation linked to micronuclei formation often occurs over several days (via overexpression of senescence-inducing oncogenic HRasV12; Dou et al., *Nature*, 2017), whereas our system operates on a rapid timescale (~16 hours) following α-synuclein exposure.

2. Mechanistic specificity

   Our data demonstrate a strong dependence on BAX-mediated MOMP, a pathway directly linked to mitochondrial permeabilization and mtDNA release (McArthur et al., *Science*, 2018; Riley et al., *EMBO J.*, 2018).

3. Functional evidence
   Pharmacological induction of MOMP using ABT-737, previously shown to promote mtDNA release (Ichim et al., *Molecular Cell*, 2015; Riley et al., *EMBO J.*, 2018), phenocopies α-synuclein effects, while inhibition of BAX suppresses both inflammation and intercellular connection formation.

4. Literature context: MN vs mtDNA
   While some studies support a role for micronuclei in cGAS activation, others indicate that:

   - Micronuclei do not consistently induce interferon responses (Flynn et al., *PNAS*, 2021)
   - DNA damage–induced micronuclei may fail to activate cGAS–STING signaling (Takaki et al., *Molecular Cell*, 2024)
   - Mitochondrial DNA release is sufficient to trigger type I interferon responses, whereas nuclear DNA damage alone may not (Tigano et al., *Nature*, 2021)

5. Neurodegeneration context
   In neurodegenerative diseases, multiple studies support a role for cytosolic mtDNA in activating cGAS–STING signaling (Yu et al., *Cell*, 2020; Matsui et al., *Nature Communications*, 2021), whereas the contribution of nuclear DNA remains less consistent and often indirect (Sharma et al., *PNAS*, 2020; Hinkle et al., *PNAS*, 2022).

Taken together, these observations support the conclusion that mitochondrial DNA represents the predominant driver of cGAS–STING activation in our experimental system.

In summary, our study provides:

- Direct evidence of cytosolic mtDNA
- Mechanistic linkage via BAX-dependent MOMP
- Functional validation through inhibition and phenocopy experiments

We therefore believe that the manuscript presents a complete and mechanistically supported study in its current form.

We have updated the discussion section of our manuscript (**in lines 491-502, also mentioned below**), highlighted in green incorporating the points raised here.

"Consistently, inhibition of BAX markedly reduced STING activation and downstream inflammatory responses, demonstrating a clear functional dependency on mitochondrial DNA release. In addition to mtDNA, nuclear DNA damage has also been reported to activate innate immune signaling. For instance, DNA damage or loss of the DNA repair kinase ATM can induce mislocalization of ssDNA to the cytosol and activate STING-dependent interferon responses[77]*. Moreover, genotoxic stress, genomic instability, and cellular senescence can promote the formation of micronuclei, which have been implicated in cGAS–STING pathway activation*[78–80]*, although their contribution remains debated*[83–85]*. In neurodegenerative contexts, nuclear DNA damage has also been linked to cGAS–STING activation, although the nature of the chromatin species involved remains unclear*[81,82]*. Future studies will be required to determine the relative contribution of nuclear versus mitochondrial DNA to cGAS–STING pathway activation in this context."*
